# Monoallelic *KRAS* (G13C) mutation triggers dysregulated expansion in induced pluripotent stem cell-derived hematopoietic progenitor cells

**DOI:** 10.1186/s13287-024-03723-2

**Published:** 2024-04-16

**Authors:** Huan-Ting Lin, Masatoshi Takagi, Kenji Kubara, Kazuto Yamazaki, Fumiko Michikawa, Takashi Okumura, Takuya Naruto, Tomohiro Morio, Koji Miyazaki, Hideki Taniguchi, Makoto Otsu

**Affiliations:** 1 Division of Regenerative Medicine, Center for Stem Cell Biology and Regenerative Medicine, Institute of Medical Science, University of Tokyo, Tokyo, 108-8639 Japan; 2 Department of Pediatrics and Developmental Biology, Graduate School of Medicine, Tokyo Medical and Dental University, Tokyo, 113-8519 Japan; 3 Tsukuba Research Laboratories, Eisai Co., Ltd., Tsukuba, Ibaraki 300-2635 Japan; 4 Department of Transfusion and Cell Transplantation, Kitasato University School of Medicine, Sagamihara, Kanagawa 252-0374 Japan; 5 Department of Regenerative Medicine, Graduate School of Medicine, Yokohama City University, Yokohama, Kanagawa 236-0004 Japan; 6 Division of Hematology, Department of Medical Laboratory Sciences, Kitasato University School of Medicine, Sagamihara, Kanagawa 252-0373 Japan

**Keywords:** Human-induced pluripotent stem cells, Hematopoietic system, Stem cells, Lymphoproliferative disorders, KRAS protein, Oncogene, Apoptosis regulatory proteins, Cell cycle, Inhibitors, Biological models

## Abstract

**Background:**

Although oncogenic RAS mutants are thought to exert mutagenic effects upon blood cells, it remains uncertain how a single oncogenic *RAS* impacts non-transformed multipotent hematopoietic stem or progenitor cells (HPCs). Such potential pre-malignant status may characterize HPCs in patients with RAS-associated autoimmune lymphoproliferative syndrome-like disease (RALD). This study sought to elucidate the biological and molecular alterations in human HPCs carrying monoallelic mutant *KRAS* (G13C) with no other oncogene mutations.

**Methods:**

We utilized induced pluripotent stem cells (iPSCs) derived from two unrelated RALD patients. Isogenic HPC pairs harboring either wild-type *KRAS* or monoallelic *KRAS* (G13C) alone obtained following differentiation enabled reliable comparative analyses. The compound screening was conducted with an established platform using *KRAS* (G13C) iPSCs and differentiated HPCs.

**Results:**

Cell culture assays revealed that monoallelic *KRAS* (G13C) impacted both myeloid differentiation and expansion characteristics of iPSC-derived HPCs. Comprehensive RNA-sequencing analysis depicted close clustering of HPC samples within the isogenic group, warranting that comparative studies should be performed within the same genetic background. When compared with no stimulation, iPSC-derived *KRAS* (G13C)-HPCs showed marked similarity with the wild-type isogenic control in transcriptomic profiles. After stimulation with cytokines, however, *KRAS* (G13C)-HPCs exhibited obvious aberrant cell-cycle and apoptosis responses, compatible with "dysregulated expansion," demonstrated by molecular and biological assessment. Increased BCL-xL expression was identified amongst other molecular changes unique to mutant HPCs. With screening platforms established for therapeutic intervention, we observed selective activity against *KRAS* (G13C)-HPC expansion in several candidate compounds, most notably in a MEK- and a BCL-2/BCL-xL-inhibitor. These two compounds demonstrated selective inhibitory effects on *KRAS* (G13C)-HPCs even with primary patient samples when combined.

**Conclusions:**

Our findings indicate that a monoallelic oncogenic *KRAS* can confer dysregulated expansion characteristics to non-transformed HPCs, which may constitute a pathological condition in RALD hematopoiesis. The use of iPSC-based screening platforms will lead to discovering treatments that enable selective inhibition of *RAS*-mutated HPC clones.

**Supplementary Information:**

The online version contains supplementary material available at 10.1186/s13287-024-03723-2.

## Background

RAS-associated autoimmune lymphoproliferative syndrome (ALPS)-like disease (RALD) is a disorder caused by a single somatic gain-of-function mutation in *KRAS* or *NRAS* genes. Its main pathologic feature is characterized by the dysregulation of immune blood cells, leading to autoimmune-like manifestations [1–3]. The same mutations are found in both myeloid and lymphoid lineage cells, indicating the origin of the causal genetic alteration at the level of an early precursor or hematopoietic stem cell capable of multi-lineage differentiation [1, 2, 4, 5]. RALD patients exhibit a clinical presentation not only similar to ALPS but also juvenile myelomonocytic leukemia (JMML) [3, 6]. Of note, a significant proportion of JMML patients are reported to share identical somatic *KRAS* or *NRAS* mutations with RALD [3]. A reported case of progression from RALD to JMML [4] and spontaneous remission of JMML leading to RALD phenotypes with persistent *RAS*-mutated clones [7] support the idea that these disorders are not distinct entities but a continuum characterized by a somatic gain-of-function *RAS* mutation in multipotent hematopoietic stem or progenitor cells (hereafter HPCs). Although RALD has initially been considered a chronic, benign disorder, recently reported its pre-malignant nature not infrequently leading to fatal outcomes necessitates a thorough understanding of the pathophysiology and the development of curative treatment [3, 4, 6].

Cellular abnormalities described to date for RALD have been observed mainly in T lymphocytes, including their impairment in Interleukin (IL)-2 depletion-induced apoptosis, also described as activated cell-autonomous death [1, 3, 4, 6, 8]. In contrast, a detailed analysis of patient marrow HPCs has yet to be reported, partly due to the limited availability of primary samples. Therefore, it remains unknown how the monoallelic oncogenic *RAS* mutation alone could impact the biological properties of human HPCs, which would lead to the RALD pathophysiology.

We previously reported the generation of induced pluripotent cells (iPSCs) carrying a single monoallelic *KRAS* (G13C) mutation from two unrelated RALD patients (Additional file 1: Table S1) [9]. We demonstrated that *KRAS* (G13C) conferred enforced retention of self-renewal upon RALD-iPSCs, an aberrant characteristic that became evident in the absence of bFGF. In the present study, we sought to extend our previous findings by scrutinizing how iPSC-derived HPCs harboring *KRAS* (G13C) showed alterations in molecular signatures and biological properties using our established hematopoietic differentiation system [10]. Due to the somatic nature of genetic mutations, *RAS*-mutated and wild-type HPCs exist in a mosaic state within the bone marrow (BM) of RALD patients. Therefore, it would be of clinical significance to clarify the differences between these two populations in cellular properties, such as proliferation and susceptibility to cell death within the given environmental conditions. Such differences identified in iPSC-HPC modeling could lead to the development of treatment modalities for RALD, enabling selective eradication of HPCs expressing oncogenic *RAS* while preserving non-mutated counterparts.

Due to global need, extensive efforts have been made as drug discovery to target oncogenic RAS in cancer research [11, 12]. Although the generation of oncogenic KRAS inhibitors targeting the G12C form has paved the way for RAS targeting therapies [13–16], acquired tumor resistance to inhibitors constitutes a drawback limiting their use as monotherapy [17, 18]. Because of these limitations, there is currently a preference for the co-inhibition of different targets to combat *KRAS*-mutant cells [19, 20]. Considering the absence of specific *KRAS* (G13C) inhibitors, we thought it rational to seek a combination of drugs with different mechanisms of action (MOA) that could effectively treat RALD at an HPC level. To enable such an approach, establishing a system capable of screening various combinations of inhibitors was necessary.

Here, we demonstrate how a single monoallelic *KRAS* (G13C) affects the molecular and biological properties of human HPCs by using iPSC-based disease modeling. With the selected culture conditions, we adopted it to become a screening platform that potentially allows the identification of drugs or combination therapies for RALD patients.

## Methods

### Chemicals

All inhibitors, including Navitoclax (BCL-2/BCL-xL), Trametinib (MEK), Palbociclib (CDK4/6), were obtained from Selleckchem.

### Patient autologous iPSCs

The characterization of iPSCs generated from two RALD patients is described [9]. The name of each iPSC clone matches that used in previous experiments [9], with the letters C and R corresponding to the control (KRAS wild-type) and the mutant [KRAS (G13C)] phenotypes, respectively (Additional file 1: Table S1). The mutant clones were confirmed for their monoallelic *KRAS* (G13C) mutation. We also used a particular pair of gene-corrected (C8) and non-edited (F4) iPSC clones; both originated from the R1-2 clone. Whole-exome sequencing did not demonstrate any other oncogenic mutations other than *KRAS* (G13C) in the tested isogenic pair of samples (Additional file 2: Table S4; Additional file 3: Table S5). Normal karyotypes were confirmed by tests performed at Nihon Gene Research Laboratories (Sendai, Japan).

### Whole exome sequencing (WES) analysis

WES analysis was performed using a standard protocol. The isogenic iPSC pairs of C8/F4 and C2-1/R2-1 were analyzed. In brief, genomic DNA was fragmented, and exonic sequences were enriched using the SureSelect Human All Exon 38 Mb kit (Agilent). Captured fragments were purified and sequenced on a Hiseq2000 platform (Illumina). Bioinformatic analysis was performed using an in-house algorithm.

### Hematopoietic differentiation and expansion

**Fig. 1:**
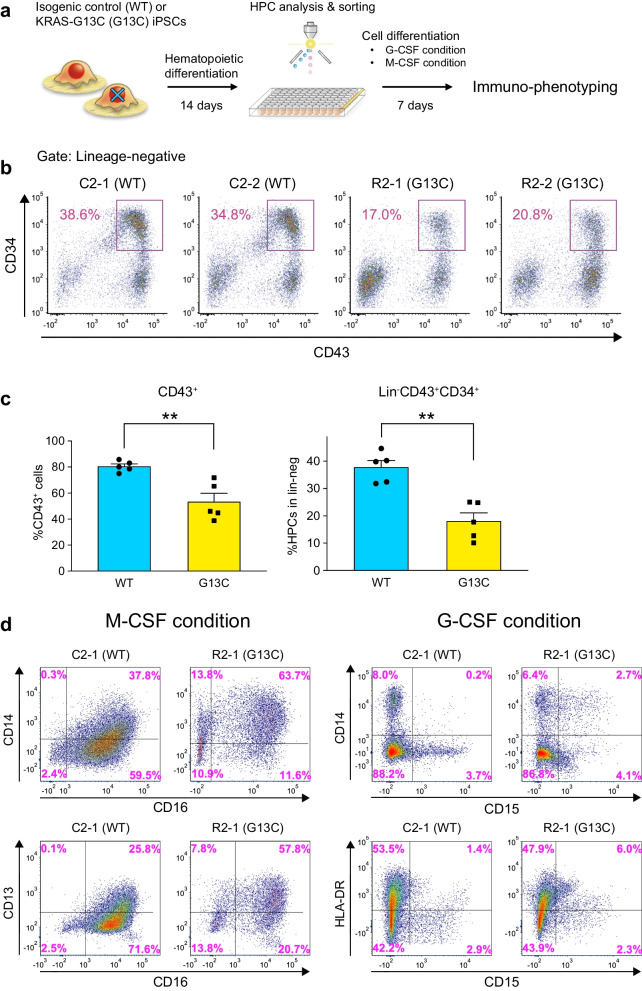
*KRAS* (G13C) affects iPSC-derived HPCs in generation efficiencies and myeloid differentiation properties. **a** A schematic overview of hematopoietic progenitor cell (HPC) and myeloid cell differentiation from isogenic RALD-iPSC pairs. After 14 days of hematopoietic differentiation followed by immunophenotypic analysis, Lin^−^CD34^+^CD43^+^ HPCs were sorted and differentiated into myeloid lineages. **b** Shown are the results obtained with two clones, either of control (WT) or mutant (G13C) iPSCs. The percentage of CD34^+^CD43^+^ events within the lineage marker-negative population is shown. **c** Comparison of percent values for the given populations between control (C2-1 and C2-2) and mutant (R2-1 and R2-2) groups. Mean ± SEM values are shown (independent experimental replicates: n = 5). Shown are the *P* values calculated in the statistical analysis based on a Mann–Whitney test. **p < *0.01*. **d** Immunophenotyping of differentiated cells directed towards a monocytic (M-CSF condition) or a granulocytic (G-CSF condition) lineage. Shown are representative plots obtained using the wild-type (WT) C2-1 and the R2-1 carrying *KRAS* (G13C) derived from patient 2

iPSCs were induced to undergo hematopoietic differentiation as previously described [10]. Briefly, human iPSCs were first differentiated into hematopoietic progenitor cells following reported procedures [3] with slight modifications. Irradiated (50 Gy) C3H10T1/2 feeder cells were co-cultured with detached iPSC colonies in hematopoietic cell differentiation medium (Iscove's modified Dulbecco's medium supplemented with 15% fetal bovine serum [FBS] and a cocktail of 10 µg/ml human insulin, 5.5 µg/ml human transferrin, 5 ng/ml sodium selenite, 2 mM L-glutamine, 0.45 mM a-monothioglycerol, and 50 µg/ml ascorbic acid in the presence of VEGF). On day 14, hematopoietic progenitor-like cells were harvested from "sac-like" structures and subjected to cell-sorting. Multipotent HPCs (Lin^−^CD34^+^CD43^+^) were sorted using the FACS Aria I flow cytometer (BD Biosciences). The resulting data was analyzed using the FlowJo software version 10.6.1 (Tree Star) or the FCS Express version 7 (De Novo Software). Sorted HPCs were differentiated into myeloid-lineage cells as described [10]. Briefly, HPCs were continuously co-cultured with irradiated C3H10T1/2 cells to differentiate into granulocytic lineage cells in the medium (αMEM + 10%FBS [Biological Industries, Kibbutz Beit Haemek, Israel] + 1% PSG) including 50 ng/ml G-CSF (R&D Systems, Minneapolis, USA) with a half medium change every 3 days (G-CSF condition in Fig. 1d). M-CSF (R&D Systems) was used for HPC differentiation into monocytic lineage cells (M-CSF condition in Fig. 1d). Expansion culture was carried out using StemSpan™-ACF (Stemcell Technologies) supplemented with either single or combinations of SCF (stem cell factor), TPO (thrombopoietin), FLT3LG (Fms-related tyrosine kinase 3 ligand), and IL-3 (all from PeproTech) as indicated.

### Quantification of cell growth

Precise cell counts were determined using Flow-Count Fluorosphere beads (Beckman Coulter) as described [21]. As shown in Figure S1a, multipotent HPCs (Lin^−^CD34^+^CD43^+^) were sorted directly into multi-well plates containing the expansion culture medium StemSpan™-ACF supplemented with either single or combinations of cytokines as indicated. Input cells were 4,000 cells per well for 48-well plates or 10,000 cells per well for 24-well plates. Seven days later, the fixed number of Flow-Count beads were added to each well. Cultured cells were harvested together with the added beads from each well and subjected to flow cytometry analysis. Viable cells were defined by the FSC/SSC characteristics and enumerated based on the event ratios of the cells to the beads. For a more rapid and efficient estimation of cell growth in a screening-compatible manner, we used CellTiter-Glo® Luminescent Viability Assay with 96-well plates (input cells = 3000/well) and the GloMax® Explorer Multimode Microplate Reader (Promega).

### RNA sequencing (RNA-seq) analysis

**Fig. 2:**
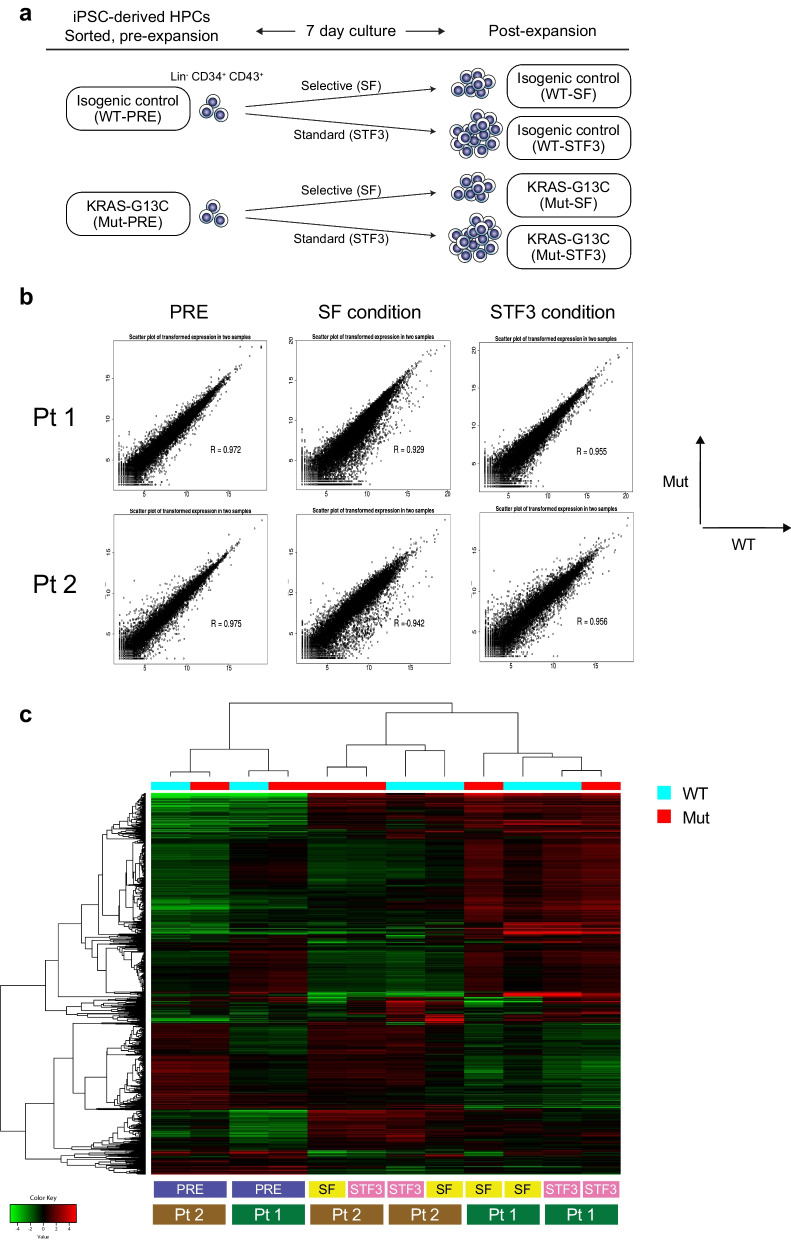
A distinct transcriptome profile induced by a single *KRAS* (G13C) in iPSC-HPCs. **a** Schematic representation of the transcriptome analysis using an RNA-sequencing method. The HPC samples derived from an isogenic iPSC pair were subjected to the analysis as pre-expansion materials (PRE). After 7-day culture, the entire population was treated as a post-expansion sample. Sample names are based on the cytokine conditions (SF: Selective, or STF3: Standard). The pair samples from both patients were used (C1-1/R1-2 and C2-1/R2-1). Overall, the 12 samples in total were subjected to the RNA-sequencing analysis. **b** Scatter plots of transformed expression values (EdgeR) generated using the iDEP program (with the 16,498 genes available after filtering). The six patterns of each pair of control (WT) and mutant (Mut) are demonstrated. **c** A heatmap showing a clustering pattern. The pattern stays virtually the same, with varied numbers of most variable genes (1000–5000) included for the analysis. Note that the profiles are highly similar between genotypes (WT vs. Mut) in PRE samples for both patients, whereas they become more distinct in post-expansion samples. Notably, the clustering occurs preferentially within the same genetic background (i.e., each patient), supporting the importance of using isogenic pairs for comparative studies

For characterization and comparison of transcriptomes, the isogenic iPSC clone pairs of C1-1/R1-2 (patient 1) and C2-1/R2-1 (patient 2) were subjected to RNA-seq analysis. In total, 12 samples were analyzed (see Fig. 2a). RNA extraction from cell pellets, library preparation, and RNA sequencing were performed by Takara-Bio. The analyses generated 150 bp (C1-1/R1-2) or 100 bp (C2-1/R2-1) pair end raw reads, of which ~ 60–100 million were mapped per sample against the human genome (GRCh37/hg19). The obtained read count data were analyzed using a web-based iDEP.93 (integrated Differential Expression and Pathway analysis, version 0.93) tool (http://bioinformatics.sdstate.edu/idep93/) [22]. The clustering DEG and pathway enrichment analyses were performed with default settings unless specified in each figure legend. Principal component analysis (PCA) was also conducted within the iDEP system.

### Real-time quantitative reverse-transcription (qRT)-PCR

Real-time qRT-PCR analysis was conducted as previously described [23]. Total RNA was isolated from iPSC-derived hematopoietic progenitor cells (HPCs) using the RNeasy® Mini Kit (Qiagen) or Monarch® Total RNA Miniprep Kit (New England Biolabs). Complementary DNA (cDNA) was synthesized using the High-Capacity cDNA Reverse Transcription Kit (Applied Biosystems). Gene expression levels were quantitated using a real-time PCR SYBR Green method with the Power SYBR Green PCR Master Mix (Applied Biosystems). G6PD was used as the reference gene. The primer sequences are from the previous report [24] and are listed in Additional file 1: Table S2. All the real-time PCR analyses were performed using a CFX96 C1000™ thermal cycler (Bio-Rad). Relative expression was calculated by the ΔΔCt method with CFX™ Manager software (Bio-Rad).

### Flow cytometry analysis

The cell cycle status of expanded cells was determined by the Click-iT™ EdU Cell Proliferation Kit (Thermo Fisher Scientific) combined with the FxCycle™ Violet Stain (Invitrogen). The cells were processed according to the manufacturer's instructions and analyzed by flow cytometry. A detailed assessment of cell death characterization was carried out with Annexin V and 7AAD used as indicators (BD Biosciences). Staining of target molecules was conducted following previously described methods [10, 25, 26]. Fluorescence intensities of these markers and expression levels of molecules were determined by flow cytometry either on the cell surface or intracellularly, as previously described [10, 26, 27]. The antibodies used are listed in Additional file 1: Table S3. Data were acquired on FACSAria™ I or II sorter (BD Biosciences) and analyzed using FCS Express 7 software (De Novo Software).

### Determining combination treatment effects on the ratio of *KRAS* G13C/WT patient-derived BM CD34^+^ cells

From the BM aspirate obtained from patient 1, CD34^+^ cells were isolated by immunomagnetic separation using the CD34 MicroBead Kit (Miltenyi Biotec) according to the manufacturer's instructions. Aliquots of 1 × 10^5^ cells were stored in liquid nitrogen using CELLBANKER® (Zenoaq) until use. After thawing, primary BM CD34^+^ cells were expanded in StemPro-34 (Thermo Fisher Scientific) supplemented with SCF, TPO, FLT3LG (all at 50 ng/ml), and IL-3 (10 ng/ml). Control cells were untreated, while treated groups were expanded in the presence of a single drug only (Navitoclax at 50 nM or Trametinib at 5 nM) or a combination of both. One week later, genomic DNA was extracted from expanded cells using the NucleoSpin Tissue extraction kit (Macherey–Nagel). Using custom probes for the detection of *KRAS* (WT or G13C; Bio-Rad), the ratio of G13C/WT alleles was estimated by ddPCR with the QX200 droplet digital PCR system (Bio-Rad) as previously described [28]*.* Allele ratios were finally converted to cell mixture ratios, considering the situation of a heteroallelic mutation existing in diploid genomes.

### An inhibitor library screening assay using iPSCs

The rationale behind the screening is to utilize the "enforced retention of self-renewal" characteristic of *KRAS* (G13C)-mutant iPSCs as previously described [9]. A schematic representation is shown as illustrated in Additional file 4: Fig. S6a. Briefly, the test iPSC clone (R1-2) was cultured in 96-well plates under feeder-free conditions in the absence of bFGF. Test compounds contained in a bioactive library (Additional file 4: Fig. S6f) were added to each well using an automatic pipettor/dispenser. Control wells contained either 0% (DMSO) or 100% (1 µM PD0325901). Four days later, the extent of pluripotency retention (%OCT4-positivity) was quantified in viable iPSCs using primary mouse anti-human OCT4 antibody (Santa Cruz) followed by secondary Alexa Fluor 555-conjugated donkey anti-mouse IgG (Molecular Probes-Invitrogen) with nuclei counterstained with Hoechst 33342 (Sigma-Aldrich). Data acquisition and analysis were conducted with IN-Cell Analyzer (GE Healthcare). Immunocytochemistry analysis comparing C1-1 and R1-2 was performed as previously described [9].

### Statistical analysis

Statistical significance and interaction (where applicable) between groups were tested using GraphPad Prism version 9 (GraphPad Software). The method used for each analysis is detailed in figure legends.

## Results

### KRAS (G13C) affects iPSC-derived HPCs in generation efficiencies and myeloid differentiation characteristics

RALD pathophysiology is defined by blood cells. Besides the dysregulated lymphoproliferation, multipotent, possibly long-lived HPCs are thought to be the point at which the *KRAS/NRAS* mutation is acquired [5, 6]. Considering HPCs as a relevant therapeutic target, we first compared their generation between the isogenic pairs, using control (C2-1 and C2-2) and the *KRAS*-mutant (R2-1 and R2-2) iPSCs (details for iPSC clones derived from two unrelated patients, Pt 1 and Pt 2, are in Additional file 1: Table S1) with our established protocol (Fig. 1a) [10]. The results showed a significant decrease in the efficiency of differentiation into multipotent HPCs (Lineage marker-negative, CD43^+^CD34^+^, hereafter "iPSC-HPCs") for the mutant cells (Fig. 1b, c). We also analyzed the myeloid differentiation of the iPSC-HPC pair C2-1 and R2-1. The immunophenotyping analysis demonstrated that iPSC-HPCs carrying *KRAS* (G13C) exhibited aberrant myeloid differentiation capabilities (Fig. 1d). Of note, some of the aberrant features reported in RALD patients were found recaptured, such as the co-expression of CD14 and CD16 in monocytes and the atypical CD14 expression in granulocytes [5]. These results demonstrate the possibility for RALD-iPSCs in modeling the abnormalities inherent to HPCs harboring the monoallelic mutant *KRAS*.

### RNA-seq analysis reveals the unique molecular signature of expanded *KRAS* (G13C)-mutant HPCs

We then examined whether *KRAS* (G13C)-mutant iPSC-HPCs exhibited distinguishable features in proliferation/survival characteristics compared with the control counterparts. To identify culture conditions suited for comparing iPSC-HPCs between genotypes in their proliferative responses, we tested the effects of four hematopoietic cytokines (SCF, TPO, FLT3L, and IL-3) in a 7-day culture (Additional file 4: Fig. S1a). When using only a single cytokine, cell viability was generally poor (evident in flow cytometry analysis, data not shown), suggesting minimal support for survival or proliferation (Single cytokine, Additional file 4: Fig. S1b). Interestingly, SCF or FLT3L alone improved the viability of mutant HPCs more compared with the control cells. When used in combination, SCF and FLT3L consistently retained cell viability to the degree that permitted further assays following expansion (SF, in "Two cytokine combinations," Additional file 4: Fig. S1b). We designated this as a "selective" culture condition, meaning the one capable of favoring the mutants' selective survival, for subsequent experiments. Considering that robust HPC expansion is a prerequisite for drug-screening purposes, other combinations were also tested (three or four cytokines). As shown, the inclusion of IL-3 generally increased yields to more than 1 × 10^6^ cells for control HPCs but resulted in consistently smaller cell numbers for mutant HPCs (Additional file 4: Fig. S1b). Because IL-3 signals are known to activate the RAS pathway through its receptor complex [29], their dysregulated activation may have negative impacts on HPC proliferation. A recently reported detrimental effect of IL-3 on stem cell function in CD34^+^ cell expansion culture [30] may also support the idea that non-physiological IL-3 signals may negatively impact human HPCs. As shown in results with all cytokine combinations that include IL-3 (IL-3, S3, F3, T3, SF3, FT3, ST3, and STF3 in Fig. S1b), this cytokine seemed to be critical to the *KRAS* (G13C)-mediated biological alterations in HPCs. Therefore, we decided to use IL-3-containing culture conditions as well for subsequent experiments. Among them, the cytokine-rich combination (STF3), which also represents the standard cocktail commonly seen for the primary HPC culture in clinical settings [30, 31], was shown to support mutant HPCs' expansion to a level sufficient for downstream applications (Additional file 4: Fig. S1b, "Full cytokine combination"). We thus decided its use as a "standard" culture condition. Consistent expansion patterns were obtained when iPSC-HPCs derived from the other isogenic pair C2-2 and R2-2 were cultured under these two cytokine combinations (data not shown). These results demonstrate that *KRAS* (G13C) iPSC-HPCs become highly distinguishable through cellular characteristics such as proliferation and/or survival according to culture conditions.

Having the defined culture conditions, we next sought to identify the unique transcriptome signature inherent to *KRAS* (G13C)-mutant HPCs. We used two different isogenic pairs, C1-1/R1-2 (Pt 1) and C2-1/R2-1 (Pt 2). The iPSC-HPCs expanded under selective (SF) or standard (STF3) conditions were subjected to RNA-sequencing (RNA-seq) analysis together with non-expanded HPCs (PRE, Fig. 2a). At first, all the data obtained from 12 samples were analyzed together on the iDEP.93 platform [22]. As shown in Fig. 2b, the scatter plots demonstrated highly similar transcriptome profiles in pre-expanded HPCs (PRE) between each isogenic pair (Pt 1 and Pt 2) of *KRAS*-wild type (WT) and mutant (Mut) samples. The profile difference became greater in expanded samples (SF and STF3). A hierarchical clustering heatmap further supported these notions (Fig. 2c). Notably, the clustering pattern clearly demonstrated the distinct transcriptome signatures depending on the genetic background (i.e., Pt 1 vs. Pt 2), despite the use of purified HPCs as test materials. Based on these observations, we next analyzed each isogenic set of samples independently (Additional file 4: Figs. S2 and S3). The heatmaps demonstrated consistent clustering patterns (Additional file 4: Figs. S2a and S3a) compared with those observed in the whole sample analysis (Fig. 2c). The principal component analysis (PCA) plots showed marked similarity in the clustering pattern for both patients, with the difference between genotypes (WT vs. G13C) becoming more apparent in cultured samples (SF and STF3) than in pre-expanded pairs (Additional file 4: Figs. S2b, S3b). K-means clustering analyses suggested the presence of differentially expressed gene (DEG) sets, which could clearly distinguish *KRAS* (G13C)-mutant cells from the WT HPCs (Additional file 4: Fig. S2c and S3c). Of note is that some clusters show remarkable differences in expression levels attributable to the genotype (WT vs. G13C) when compared with each cultured pair (ST and STF3) being considered as a group (clusters B and E in Additional file 4: Fig .S2c; clusters D–F in Additional file 4: Fig. S3c). The gene sets contained in these clusters were found enriched in several biological pathways, including those related to inflammation, cell activation, myeloid differentiation, and cytokine-mediated signals (Additional file 4: Figs. S2d, e; S3d, e).

### Differentially expressed genes identified in *KRAS* (G13C)-mutant iPSC-HPCs suggest dysregulation in the cell cycle and apoptosis machinery

**Fig. 3:**
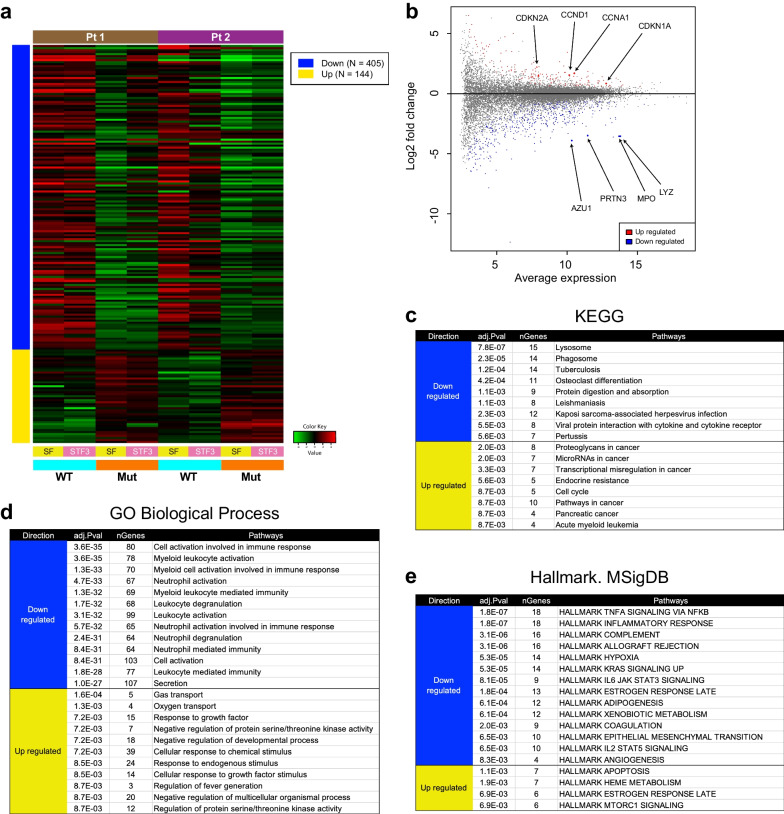
Identification and characterization of differentially expressed gene sets in *KRAS* (G13C) iPSC-HPCs. **a** A heatmap demonstrating a set of differentially expressed genes (DEG) between wild-type (WT) and *KRAS*-mutant (Mut) HPCs after 7-day culture. Data from each patient are clustered separately (Pt 1 and Pt 2). Two samples cultured with different cytokine conditions (SF and STF3) are treated as a pair within each cell type. A cut-off value of an FDR (false-discovery rate) < 0.1 and a minimal log2 fold change of 1.5 are used. The gene sets found down-regulated in Mut groups are shown at the top, whereas those up-regulated are at the bottom. **b** An MA plot depicting DEGs. Several genes of interest are indicated with the enlarged symbols in both up-regulated (red)- and down-regulated (blue)-gene sets. **c**–**e** Enrichment analysis for the DEGs in specified gene sets. Shown are adjusted *P* values (adj. *P* val) and the number of genes (nGenes) contained in each pathway group. The pathway databases used are KEGG (**c**, Kyoto Encyclopedia of Genes and Genomes), GO (Gene Ontology), Biological Process (**d**), and Hallmark. MsigDB (**e**, The Molecular Signatures Database)

**Fig. 4:**
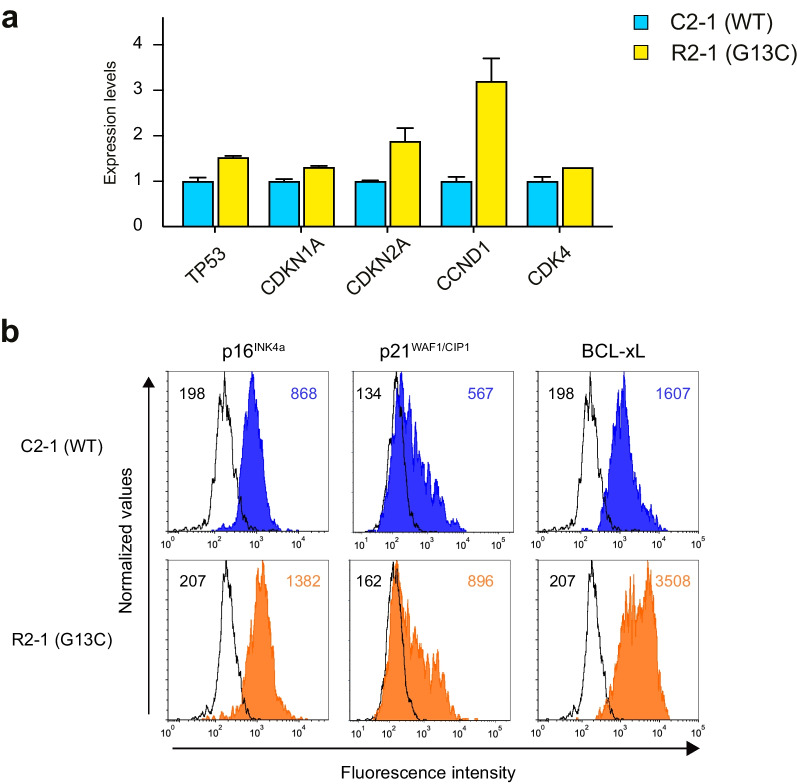
Altered gene and protein expression of cell cycle and apoptosis-related molecules in *KRAS* (G13C) iPSC-HPCs. **a** Quantitative RT-PCR analysis of selected genes related to the regulation of cell cycle and apoptosis in expanded HPCs under standard conditions. The representative results obtained with the isogenic pair C2-1 (WT) and R2-1 (G13C) in two independent experiments are presented as mean ± SEM from biological duplicate samples. **b** Intracellular flow cytometry analysis for the expression levels of indicated molecules in cultured HPCs. Shown are the representative overlay plots for C2-1 and R2-1 samples. Mean fluorescent values are shown for each histogram

We then sought to identify the DEGs directly attributable to *KRAS* (G13C). For this objective, the DEG analysis was conducted by the iDEP using only cultured HPC samples combining two patients' data (8 samples in total). With this combined analysis, we successfully picked up the 549 DEGs, of which the 405 and 144 genes were labeled as "Down-regulated" and "Up-regulated" in the mutant, respectively (Fig. 3a). The MA plot illustrated these DEGs more evidently. The pathway enrichment analysis of the identified DEGs suggested deviations in several key biological features in *KRAS* (G13C)-mutant HPCs (Fig. 3b–e). As shown, the genes found enriched in the DEG cluster contained the cell cycle-related genes (*CCNA1*, *CCND1*, *CDKN1A*, and *CDKN2A*) as "Up regulated", and the genes associated with myeloid cell maturation/function (*AZU1*, *PRTN3*, *MPO*, and *LYS*) as "Down regulated" (Fig. 3b). Enrichment was also noted in the apoptosis-related pathway for the up-regulated genes (Fig. 3e). Dysregulation in both cell cycle and apoptosis machinery in expanding mutant iPSC-HPCs was further supported by quantitative reverse transcription (qRT)-PCR analysis (Fig. 4a, Additional file 4: Fig. S4a, c). Flow cytometry analysis confirmed the increased expression of p16^INK4a^ (*CDKN2A*) and p21^WAF1/CIP1^ (*CDKN1A*) at a protein level in mutant HPCs (Fig. 4b, Additional file 4: Fig. S4b, d). Interestingly, we could also identify the increased expression of the anti-apoptotic protein BCL-xL (*BCL2L1*) as a consistent phenotype for the cultured *KRAS* (G13C)-mutant HPCs (Fig. 4b, Additional file 4: Fig. S4b, d). These results support the idea that *KRAS* (G13C) alone is sufficient to trigger the alteration in differentiation propensity and aberrations in cellular machinery critical for cell cycle and apoptosis regulation in expanding HPCs.

### Alterations in cell cycle status and cell death responses conferred on HPCs by the *KRAS* (G13C) mutation

**Fig. 5:**
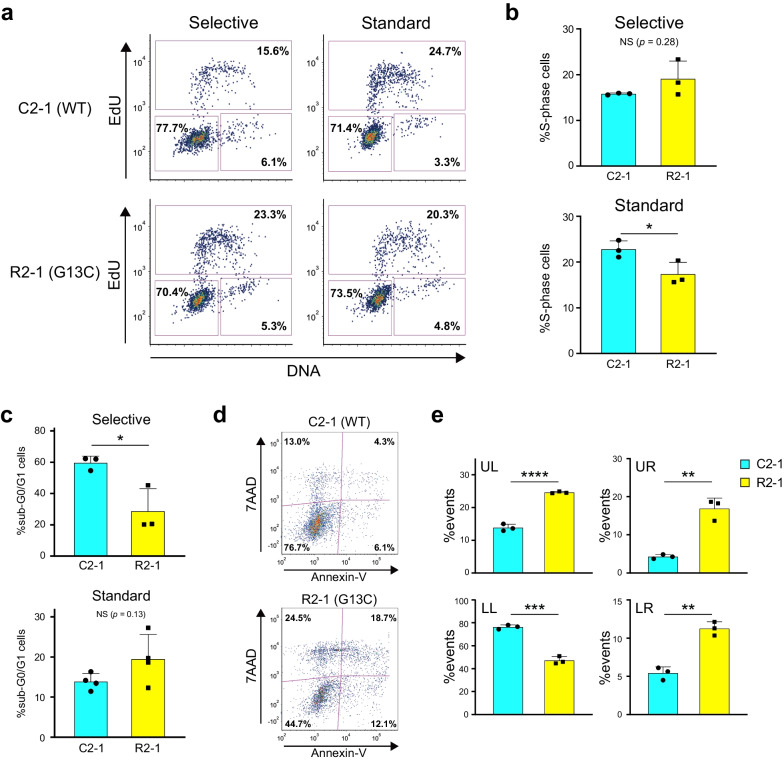
Aberrancies in cell-cycle status and apoptosis in cultured *KRAS* (G13C) iPSC-HPCs. **a** Flow cytometry-based cell-cycle analysis of control (C2-1) and mutant (R2-1) iPSC-HPCs expanded under either the SF (Selective) or STF3 (Standard) condition. Representative plots are shown with the estimated percent values of each cell-phase. G1-phase, lower left; S-phase, top; G2/M-phases, lower-right. **b** Quantitative comparison between C2-1 and R2-1 HPCs (cell percentage) in S-phase. Mean ± SD values are shown (independent experimental replicates: n = 3). **c** Cell viability analysis of C2-1 and R2-1 HPCs expanded with indicated culture conditions. With the samples processed for the cell cycle assessment, the events appearing as a distinct population characterized by a reduced DNA content (low fluorescent intensity with the FxCycle™ Violet Stain) were visualized and quantified as sub-G0/G1 cells using flow cytometry. Mean ± SD values are shown (independent experimental replicates: n = 3, Selective; n = 4, Standard). NS, not significant. **d** Detailed cell death analysis of C2-1 and R2-1 HPCs cultured in the standard condition. Representative images are shown in two-dimension plots with Annexin-V and 7AAD used as cell-death indicators. The estimated percentage for the events contained in each quadrant is indicated. **e** Quantitative comparison between C2-1 and R2-1 iPSC-HPCs in percentages of the cells in each quadrant. Mean ± SD values are shown (independent experimental replicates: n = 3). All statistical analyses are based on an unpaired t test. *p < 0.05, **p < 0.01, ***p < 0.001, ****p < *0.0001*. NS, not significant

To further clarify the unique characteristics conferred on mutant cells by *KRAS* (G13C), we performed a series of biological assays. Consistent with previous HPC culture assays (Additional file 4: Fig. S1), cell cycle analysis revealed that "selective" conditions favored the expansion of mutant iPSC-HPCs (higher percentage of mutant cells in S-phase), whereas the opposite was true for "standard" conditions (Fig. 5a, b). Since it has previously been reported that sustained oncogene activation through replicative stress induces either cell death or acquisition of senescence characteristics [32], we investigated how cultured mutant iPSC-HPCs differed from the control cells in cell death status. Consistent with results in the expansion assays and cell cycle analysis, we discovered a lower sub-G0/G1 rate (representing an apoptotic state) for mutant cells under the "selective" condition, suggesting that *KRAS* (G13C) conferred survival advantages on iPSC-HPCs in the cytokine-poor setting (Fig. 5c, Selective). Under the "standard" condition, however, cultured mutant HPCs tended to yield more dead/dying cells than their control counterparts; this was evidenced by an increase in the sub-G0/G1 fractions (Fig. 5c, Standard) or the population containing apoptotic and necrotic cells (Fig. 5d, e). These culture condition-dependent variations in susceptibility to cell death were partly supported by the analysis of signaling molecules (Additional file 4: Fig. S5). With the "selective" condition, mutant HPCs displayed molecular signatures consistent with enhanced signaling (Additional file 4: Fig. S5a), whereas they became obscure under the "standard" condition (Additional file 4: Fig. S5b). Similarly, mutant iPSC-HPCs exhibited enhanced expression of cKit, the receptor for SCF, especially with the "selective" condition (Additional file 4: Fig. S5c). These results demonstrate that the acquired *KRAS* (G13C) alone has the potential to trigger HPC dysregulation in cell cycle and cell death responses, which can be affected drastically by the varying signaling cues.

### Drug identification by selective activity to reverse KRAS (G13C)-mediated cellular changes

One remarkable advantage of the iPSC-based disease modeling is its use as a drug discovery platform, as exemplified in recent reports, including those studying hematopoietic malignancies [33–35]. To look for drugs capable of reversing the mutant *KRAS*-mediated cellular changes, we first sought to utilize the unique aberrant phenotype, enforced self-renewal, explicitly found in RALD-iPSC clones harboring the *KRAS* mutation [9]. The results from screening a 906 bioactive compound library are shown in Additional file 4: Fig. S6 (refer to the legend for details). In brief, we found 40 "hit" reagents, of which a representative reagent with known MOA from each class was subjected to the second-step screening assay using iPSC-HPCs. It was remarkable that the inhibition of two major pathways downstream of KRAS activation showed a sharp contrast in the outcomes. A PI3K inhibitor failed to lower the OCT4 expression without compromising iPSC viability, whereas a MEK inhibitor yielded a typical "hit" pattern (Additional file 4: Fig. S6e).

**Fig. 6:**
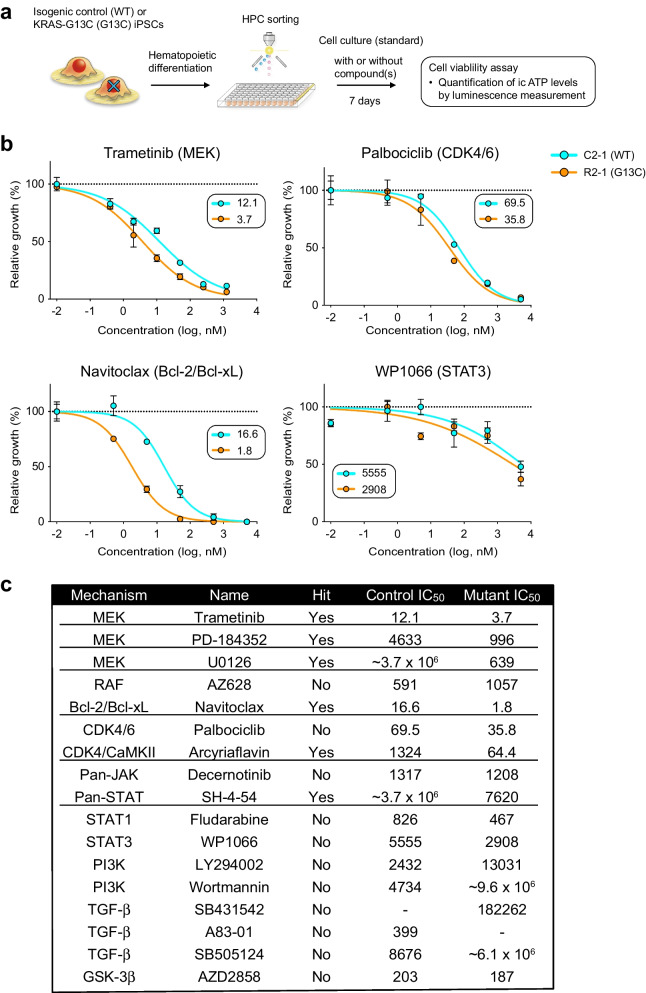
Construction of a drug screening platform capable of exploring inhibitory effects selective on *KRAS* (G13C) iPSC-HPCs. **a** A schematic representation of a drug screening system utilizing iPSC-HPCs. An isogenic pair of control (WT) and mutant (G13C) iPSC-HPCs were directly sorted into 96-well plates (n = 3 as technical replicates) and cultured for one week under the standard (unless stated) condition with or without the presence of candidate compounds. Cell viability was quantified by assessing intracellular ATP levels (ic ATP) with a luminescence measurement system. **b** Dose–response plots assessing growth inhibitory effects of a single reagent on either control (C2-1, blue) or *KRAS*-mutant (R2-1, orange) HPCs. The percent growth was estimated relative to the control values (DMSO). Calculated IC_50_ values are indicated. Shown are the examples of plots exhibiting a visible selective activity (Trametinib and Navitoclax), only modest selectivity (Palbociclib), and no such selectivity (WP1066). **c** Summary of the results obtained with candidate compounds tested in the established screening platform. The name of each compound is shown, along with its target (Mechanism) and the estimated values of IC_50_. The compounds showing reduced IC_50_ values for mutant HPCs (< 0.5 of the control IC_50_) are considered selective and thus a "Hit" compound (underlined)

We then tested whether drug screening was feasible using the iPSC-HPC culture system supplemented with the defined cytokine cocktails. To enable fast and efficient procedures, we adopted multi-well plate culturing followed by luminescence-based measurement of intracellular ATP levels (Fig. 6a). Using this platform, we sought to examine if particular drugs were capable of limiting the expansion of *KRAS-*mutant HPCs more efficiently than the control cells. During assay optimization, we observed such selective inhibition patterns with MEK-inhibitors but not with PI3K-inhibitors under both "selective" and "standard" conditions (Additional file 4: Fig. S7a). Since the large inter-assay variability could not be overcome due to poor cell viability with the "selective" condition, we decided to use the "standard" condition hereafter for all screening experiments. We selected candidate compounds based on iPSC-screening results and the *KRAS*-mutant HPCs' features characterized by dysregulation in the cell cycle and cell death machinery. We included the following inhibitors (MOA specified), Trametinib (MEK), Palbociclib (CDK4/6), and Navitoclax (BCL-2/BCL-xL) as candidate compounds, each of which was already in clinical use [36–38]. As shown in Fig. 6b, these three compounds exhibited visible selective inhibitory activity against mutant HPCs, while other compounds like WP1066 (STAT3) did not. A summary of all compounds verified by HPC screening is shown (Fig. 6c). Although these results demonstrated the feasibility and utility of the established HPC-screening assay, a narrow therapeutic window observed with these "hit" compounds was considered unacceptable for actual treatment use. In fact, the same drugs tested on another isogenic pair samples (C1-1 and R1-2) demonstrated visible selective inhibition of the mutant iPSC-HPCs only with Navitoclax (Additional file 4: Fig. S7b). Therefore, we next tested whether a combination of drugs could enhance selective effects against *KRAS* (G13C) iPSC-HPCs while preserving the viability of healthy counterparts.

### Concurrent MEK and BCL-2/BCL-xL inhibition enhances selective inhibitory effects on KRAS-mutant HPCs

**Fig. 7:**
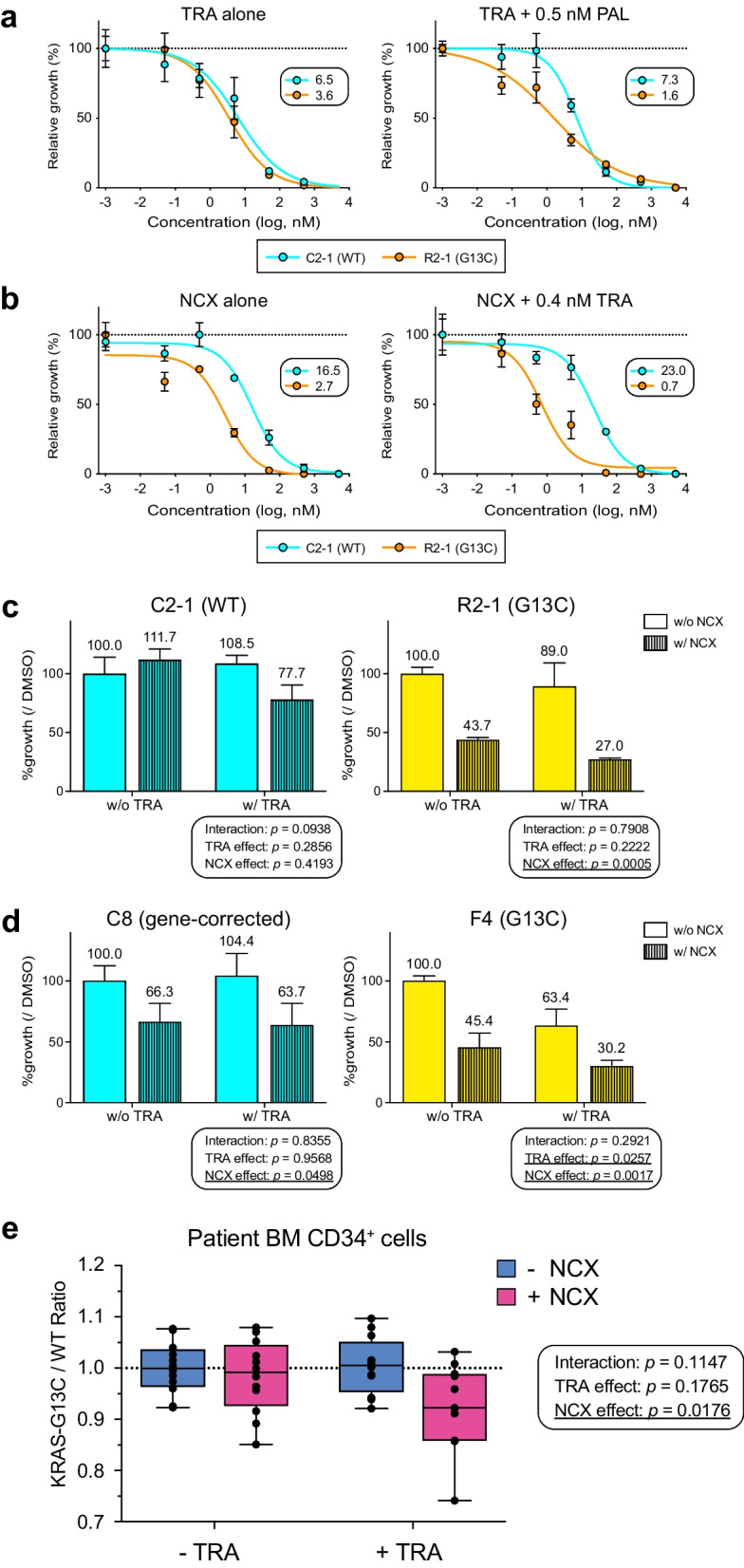
Potential enhancement of selective inhibitory effects on *KRAS* (G13C) HPCs by combination treatment. **a, b** Comparison of growth inhibitory effects between single- and combined treatments. Shown are the dose–response plots assessing treatment effects on either control (C2-1, blue) or *KRAS*-mutant (R2-1, orange) HPCs (technical replicates: n = 3). (**a**) The IC_50_ value for R2-1 samples gets lower (1.6 nM) in the presence of 0.5 nM Palbociclib (PAL) compared with that obtained with Trametinib alone (3.6 nM). In contrast, the values stay similar for C2-1 samples (6.5 nM with TRA alone and 7.3 nM in combination). (**b**) Lowered IC_50_ values observed for Navitoclax treatment with the addition of Trametinib (+ 0.4 nM TRA) in R2-1 samples (2.7 nM vs. 0.7 nM). The two values do not differ significantly for C2-1 (16.5 nM with NCX alone and 23.0 nM in combination). **c, d** Two-way analysis of variance (ANOVA) tests assessing the combination effects on iPSC-HPCs with the fixed concentration for each drug (TRA, 0.4 nM; NCX, 5 nM). Shown is the mean ± SD (technical replicates: n = 3) with the mean values provided on top of each histogram. Three *P* values are shown for each graph with underlines placed where they reach the statistical significance (< 0.05). Overall, we detected no remarkable interaction between the two main effects (TRA and NCX) and performed no post-hoc tests. (**c**) Results of the isogenic pair iPSC-HPCs derived from patient 2 (C2-1 and R2-1). (**d**) Results of the isogenic pair iPSC-HPCs derived from patient 1 (C8, a genetically corrected clone originated from the mutant iPSC R1-2; F4, a non-edited counterpart clone). Note that the combination treatment shows marked inhibitory effects on the mutant HPCs (27.0% for R2-1 and 30.2% for F4) while allowing limited toxicity to the control cells (viability of 77.7% for C2-1 and 63.7% for C8). **e** BM CD34^+^ cells isolated from RALD Pt 1 were tested. The pre-culture assessment revealed that the patient HPCs consisted of a ~ 1: 1 mixture of WT and *KRAS*-G13C cells. Shown is a box and whiskers plot comparing the estimated *KRAS*-G13C/WT ratio in each group of cells following a 7-day culture (technical replicates: n = 12. Recent death of the patient limits repetitive assessment with the primary samples). The decrease in ratio values is considered consistent with the selective inhibition of mutant cells over the control. The *P* values are based on the 2-way ANOVA. The analysis proved no interaction between the two effects, with only the inhibitory effect of navitoclax (NCX) reaching statistical significance (underlined). Despite the lack of interaction, it seems that a sensitizing effect of trametinib (TRA) exists selectively for the primary HPCs carrying *KRAS*-G13C on NCX-mediated growth inhibition.

We tested if adding a second drug at small doses could lower the IC_50_ values achieved following primary drug-mediated inhibition. The assays using the isogenic pair C2-1 and R2-1 demonstrated such a selective decrease in IC_50_ values for mutant HPCs when Trametinib was combined with 0.5 nM Palbociclib (Fig. 7a) and when Navitoclax was combined with 0.4 nM Trametinib (Fig. 7b). We observed a similar beneficial increase in the enhancement of inhibitory effects by the drug combinations when tested with the other isogenic iPSC pair (Additional file 4: Fig. S7c, d). Considering the increased BCL-xL expression in *KRAS*-mutant HPCs (Fig. 4b, Additional file 4: S4b, d) and its clinical use for lymphoid malignancies [39, 40], we sought to investigate the effects of Navitoclax for more details. Trametinib was used in combination as a direct modulator of the KRAS-downstream pathway. In experiments where fixed concentrations were adopted for each drug (TRA, 0.4 nM; NCX, 5 nM), mutant HPC viability could be reduced to significantly lower levels (27.0%, R2-1), whereas control cell viability remained well preserved (77.7%, C2-1) (Fig. 7c). Similar observations were obtained by testing HPCs derived from another isogenic iPSC pair (Pt 1), comprised of gene-corrected (C8) and uncorrected (F4) clones (Fig. 7d). Although the synergism was not proved between the two drugs by statistical analyses, these results demonstrated possible sensitizing effects for one drug to another, which would favor selective inhibition of *KRAS* (G13C)-mutant iPSC-HPCs.

Lastly, we examined patient-derived BM CD34^+^ cells (Pt 1), using ddPCR analysis to determine the ratio of G13C/WT KRAS cells. Pre-expansion, the ratio was confirmed to be approximately one, indicating a roughly 50% distribution of each fraction. After a 1-week expansion with the cytokines, the ratio remained unchanged in the untreated group (Fig. 7e, -TRA, -NCX). With a single compound treatment, the overall cell recovery declined (data not shown), but the G13C/WT ratio remained unaltered, meaning no selective inhibition forced on one population (-TRA/ + NCX, + TRA/-NCX). In contrast, the combination treatment led to a visible decrease in the ratio, suggesting enhanced selective inhibition targeting mutant cells in this clinically relevant model. Overall, these results show the possibility of developing strategies that enhance the selective inhibitory activity against *KRAS* (G13C)-mutant HPCs while potentially broadening the therapeutic window through optimal combinations of drugs.

## Discussion

Here, we reported detailed comparative analyses of HPCs that were differentiated from RALD patient-derived iPSCs. Compared with iPSC-based modeling of hematologic malignancies that typically contain multiple mutated oncogenes [33, 41, 42], our RALD-iPSCs acted as an invaluable tool to investigate the considerable effect of *KRAS* (G13C) on cellular functions within an isogenic HPC pair. Besides studies examining the impact of heterozygous mutation of *NRAS* (G12D) [43] or *KRAS* (G12D) [44, 45] for murine HPCs, to the best of our knowledge, this is the first study to analyze the effects of an oncogenic RAS protein expressed under endogenous control in the context of non-immortalized, human diploid HPCs. Recently, the review paper reported a variation of *RAS* mutations in 27 RALD patients, showing eight cases for both *KRAS* (G13C) and *NRAS* (G13D) as the most frequent genotypes, followed by three cases of *KRAS* (G13D) [3]. Although six cases showed codon 12 mutations, only one patient had *KRAS* (G12D) [2, 5], with others displaying distinct amino acid substitutions [G12A or G12S for *KRAS*, and G12S and G12V for *NRAS*]. For JMML, it is reported that *KRAS* (G13D) and *NRAS* (G12D) are the predominant mutations [46]. Considering a significant level of variation in *RAS* mutations in blood disorders, comparative studies are warranted to test each mutant for its roles in HPC pathogenesis using the defined experimental models, including transgenic mice and iPSC-HPCs harboring monoallelic *RAS* mutations of interest.

The biological effects of KRAS (G13C) expression on iPSC-HPCs appeared to depend on the context of external stimuli. Induction to myeloid lineages revealed significantly altered differentiation properties in mutant HPCs compared with those in control cells, mimicking, in part, the abnormalities reported in RALD patients [5]. RNA-seq analysis identified a group of myeloid-specific genes being DEG, supporting the idea that *KRAS* (G13C) significantly impacts HPCs' differentiation capabilities. In expansion cultures, the minimal cytokine condition ("selective") favored survival for *KRAS* (G13C)-HPCs over the control. This feature may mimic the gradual clonal expansion of mutant HPCs that likely occurs within patient BM at an early latent phase. Stimulation with "standard" cytokines yielded contrasting outcomes, as mutant HPCs showed more dead cells with lower expansion rates than control cells. Molecular analysis of surviving mutant HPCs revealed notable changes, including increased expression of anti-apoptotic BCL-xL, and CDK inhibitors p16^INK4a^ (*CDKN2A*) and p21^WAF1/CIP1^ (*CDKN1A*). The induction of these molecules is consistent with senescence phenotypes, known to be brought by oncogenic RAS expression [47, 48]. Although the higher death rates induced for mutant HPCs may contradict the increased BCL-xL expression, this may simply reflect the heterogeneous nature of HPCs cultured in the standard condition. Since the in-depth understanding of fate decisions of HPCs at a single cell level has yet to be acquired under stressed conditions [49, 50], studies are needed to elucidate further details about the dysregulation of cell cycle and apoptosis machinery observed in *KRAS* (G13C)-mutant iPSC-HPCs.

Notably, we used iPSCs originated from two unrelated patients carrying *KRAS* (G13C). Comprehensive RNAseq analyses confirmed the value of isogenic pairs, allowing the clarification of molecular signatures commonly altered in HPCs attributable to *KRAS* mutation across genetic backgrounds. It has long been an issue of debate in the stem cell field whether the heterogeneity of iPSC clones can be attributed to genetic variations, cell sources, and generation methods [51–54]. Our Sendai virus-based protocol allowed the simultaneous establishment of paired isogenic iPSC clones using a single CD34^+^ cell aliquot obtained from RALD patients [23, 55]. This likely contributed to the striking similarity found in transcriptome profiles between pre-expanded iPSC-HPC pair samples of the same donor origin despite their difference in *KRAS* status. Such minimum variability achievable by amended procedures is considered mandatory in iPSC-based disease modeling to enable reliable comparative analysis.

*RAS* mutations, including those affecting KRAS at codon 12 or 13, are frequently identified in many cancer types [11, 12]. Despite ongoing efforts to directly target oncogenic RAS proteins, progress has been hampered by their unique "undruggable" nature [56, 57]. In this study, we demonstrated the feasibility and usefulness of a screening system using HPCs derived from RALD-iPSCs harboring *KRAS* (G13C). Although still preliminary, our study identified Navitoclax combined with Trametinib as a possible combination benefiting RALD patients. Further improvement of the platform will allow high-throughput screening of compounds alone or in combinations, leading to identifying curative treatments for RALD. The recent approval of a BCL-2 inhibitor venetoclax in combination therapy for myeloid malignancies likely justifies this direction of drug discovery [58, 59]. As stated earlier, no single RALD patients have been reported to possess the *KRAS* (G12C) genotype. Also, other hematologic malignancies are reportedly associated with *KRAS* (G12D) or *KRAS* (G13D), not with the *KRAS* (G12C) mutation [60]. Therefore, it is desirable to develop a new target-specific RAS inhibitor other than the one targeting KRAS (G12C), which is applicable to intractable hematopoietic malignancies. Because our study proposes the feasibility of a similar system establishment using iPSCs combined with genome-editing techniques, more efforts will eventually enable us to extend the research to a broader range of RAS oncogenes, culminating in the conversion of undruggable RAS to druggable molecules.

## Conclusions

This study demonstrated that a monoallelic oncogenic *KRAS* alone could confer dysregulated expansion characteristics to non-transformed HPCs. To the best of our knowledge, this is the first report formally addressing how a monoallelic oncogenic RAS affects non-immortalized, human diploid HPCs with its expression regulated under endogenous control. We believe that these results have implications not only for RALD pathophysiology but also for RAS basic biology.

### Supplementary Information

**Additional file 1. Table S1.** Information on RALD patients and iPSC clones. **Table S2.** Primer list used for qPCR assays. **Table S3.** Antibody list.

**Additional file 2. Table S4.**

**Additional file 3. Table S5.** Whole exome sequencing analysis data of isogenic pair samples of iPSC clones (Table S4: C8 and F4, Pt 1; Table S5: C2-1 and R2-1, Pt 2).

**Additional file 4. Fig. S1.** Comprehensive screening of cytokine combinations to elucidate the effect of *KRAS (G13C)* mutation on iPSC-HPCs. **Fig. S2.** Characterization of altered transcriptome profiles brought by a single *KRAS (G13C)* mutation in iPSC-derived HPCs (patient 1 samples). **Fig. S3.** Characterization of altered transcriptome profiles brought by a single *KRAS (G13C)* mutation in iPSC-derived HPCs (patient 2 samples), related to Figs. 2 and 3. **Fig. S4.** Altered gene and protein expression of cell-cycle and apoptosis-related molecules in *KRAS*-mutant HPCs, related to Fig. 4. **Fig. S5.** Aberrancies in cell signaling pathways and cKit expression conferred on cultured iPSC-derived HPCs carrying the *KRAS (G13C)* mutation, related to Fig. 5. **Fig. S6.** Inhibitor library screening to identify effective modulators of aberrant KRAS signaling in iPSCs. **Fig. S7.** Utilization of the established drug screening platform to explore inhibitory effects significantly selective on* KRAS*-mutant HPCs, related to Figs. 6 and 7.

## Abbreviations

ALPS: Autoimmune lymphoproliferative syndrome
BM: Bone marrow
DEG: Differentially expressed gene
FLT3LG: Fms-related tyrosine kinase 3 ligand
HPCs: Hematopoietic progenitor cells
iDEP.93: Integrated differential expression and pathway analysis, version 0.93
IL: Interleukin
iPSCs: Induced pluripotent stem cells
MOA: Mechanisms of action
PCA: Principal component analysis
RALD: RAS-associated autoimmune lymphoproliferative syndrome-like disease
RNA-seq: RNA sequencing
SCF: Stem cell factor
TPO: Thrombopoietin
WES: Whole exome sequencing

## Declarations

### Availability of data and materials

RNAseq data are deposited in the NCBI GEO under the code GSE200600. Other data sets are available from the corresponding author upon request.
