# Supplementary material for: Monoallelic KRAS (G13C) mutation triggers dysregulated expansion in induced pluripotent stem cell-derived hematopoietic progenitor cells
Source: Stem Cell Res Ther. 2024 Apr 16;15:106. doi: 10.1186/s13287-024-03723-2 (PMC11021011; doi:10.1186/s13287-024-03723-2)
Supplement: Supplementary file 1 — Additional file 1. Table S1. Information on RALD patients and iPSC clones. Table S2. Primer list used for qPCR assays. Table S3. Antibody list. [file 13287_2024_3723_MOESM1_ESM.pdf]

Table S1. Information of RALD patients and iPSC clones (Kubara *et al*., Stem Cell Reports 2018;11:380-394. doi: 10.1016/j.stemcr.2018.06.008.)

Patient	Mutation	*KRAS* genotype	Clone names	Description
Patient 1	G13C (GGC > TGC)	WT/WT	C1-1	Isogenic pair clones established from the same batch of BM CD34$^+$ cells
		WT/G13C	R1-2	
		WT/WT	C8*	Isogenic pair clones obtained by genome-editing the clone R1-2: C8, corrected; F4, uncorrected
		WT/G13C	F4*	
Patient 2	G13C (GGC > TGC)	WT/WT	C2-1**	Isogenic pair clones established from the same batch of BM CD34$^+$ cells
		WT/G13C	R2-1**	
		WT/WT	C2-2	Isogenic pair clones established from the same batch of BM CD34$^+$ cells
		WT/G13C	R2-2	

* These two samples were subjected to whole exome analysis (shown in Table S2).

** These two samples were subjected to whole exome analysis (shown in Table S3).

Table S2. Primer list

Genes	Abbreviation	Forward (5’ -> 3’)	Reverse (5’ -> 3’)	Size (bp)
Tumor protein p53	*TP53*	TGCAGCTGTGGGTTGATTCC	AAACACGCACCTCAAAGCTGTTC	396
Cyclin-dependent kinase inhibitor 1A (p21, Cip1)	*CDKN1A*	CCTCATCCCGTGTTCTCCTTT	GTACCACCCAGCGGACAAGT	97
Cyclin-dependent kinase inhibitor 2A (p16, Ink4A)	*CDKN2A*	CAACGCACCGAATAGTTACGG	AACTTCGTCCTCCAGAGTCGC	96
Cyclin D1	*CCND1*	ACGAAGGTCTGCGCGTGTT	CCGCTGGCCATGAACTACCT	323
Cyclin-dependent kinase 4	*CDK4*	CTGGTGTTTGAGCATGTAGACC	AAACTGGCGCATCAGATCCTT	102
Glyceraldehyde-3-phosphate dehydrogenase	*GAPDH*	TGCACCACCAACTGCTTAGC	GGCATGGACTGTGGTCATGAG	87

Table S3. Antibody list

Antibody	Clone	Host/Isotype	Conjugation	Supplier
Anti-CD13	WM15	Mouse / IgG1	FITC	Abcam
Anti-CD14	61D3	Mouse / IgG1, kappa	PE/Cyanine7	eBioscience
Anti-CD15	W6D3	Mouse / IgG1, kappa	BV605	Biolegend
Anti-CD16	B73.1	Mouse / IgG1, kappa	APC	Biolegend
Anti-CD235ab	HIR2	Mouse / IgG2b, kappa	Pacific Blue	Biolegend
Anti-CD34	581	Mouse / IgG1, kappa	PE/Cyanine7	Biolegend
Anti-CD41a	HIP8	Mouse / IgG1, kappa	FITC	BD Pharmingen
Anti-CD43	CD43-10G7	Mouse / IgG1, kappa	APC	Biolegend
Anti-CD45	HI30	Mouse / IgG1, kappa	APC/Cyanine7	Biolegend
Anti-HLA-DR	L243	Mouse / IgG2a, kappa	PerCP	Biolegend
Anti-p16 INK4a/CDKN2A	EP435Y-129R	Rabbit / IgG	Purified	Abcam
Anti-p21 Waf1/Cip1/CDKN1A	F-5	Mouse / IgG2b, kappa	Purified	Santa Cruz
Anti-Bcl-xL	54H6	Rabbit / IgG	Purified	Cell Signaling Technology
Anti-pAKT	M89-61	Mouse / IgG1, kappa	PE	BD Biosciences
Anti-pERK1/2	20A	Mouse / IgG1	PE	BD Biosciences
Anti-pSTAT3	49/p-Stat3	Mouse / IgG1	PE	BD Biosciences
Anti-Rabbit IgG	Polyclonal	Rabbit / IgG	Alexa Fluor 488	ThermoFisher
Anti-Mouse IgG	Polyclonal	Mouse / IgG	Alexa Fluor 488	ThermoFisher
Anti-Mouse IgG	MOPC-21	Mouse / IgG1, kappa	PE	BD Biosciences
