# Supplementary material for: Monoallelic KRAS (G13C) mutation triggers dysregulated expansion in induced pluripotent stem cell-derived hematopoietic progenitor cells
Source: Stem Cell Res Ther. 2024 Apr 16;15:106. doi: 10.1186/s13287-024-03723-2 (PMC11021011; doi:10.1186/s13287-024-03723-2)
Supplement: Supplementary file 4 — Additional file 4. Fig. S1. Comprehensive screening of cytokine combinations to elucidate the effect of KRAS (G13C) mutation on iPSC-HPCs. Fig. S2. Characterization of altered transcriptome profiles brought by a single KRAS (G13C) mutation in iPSC-derived HPCs (patient 1 samples). Fig. S3. Characterization of altered transcriptome profiles brought by a single KRAS (G13C) mutation in iPSC-derived HPCs (patient 2 samples), related to Figs. 2 and 3. Fig. S4. Altered gene and protein expression of cell-cycle and apoptosis-related molecules in KRAS-mutant HPCs, related to Fig. 4. Fig. S5. Aberrancies in cell signaling pathways and cKit expression conferred on cultured iPSC-derived HPCs carrying the KRAS (G13C) mutation, related to Fig. 5. Fig. S6. Inhibitor library screening to identify effective modulators of aberrant KRAS signaling in iPSCs. Fig. S7. Utilization of the established drug screening platform to explore inhibitory effects significantly selective on KRAS-mutant HPCs, related to Figs. 6 and 7. [file 13287_2024_3723_MOESM4_ESM.pdf]

Figure S1

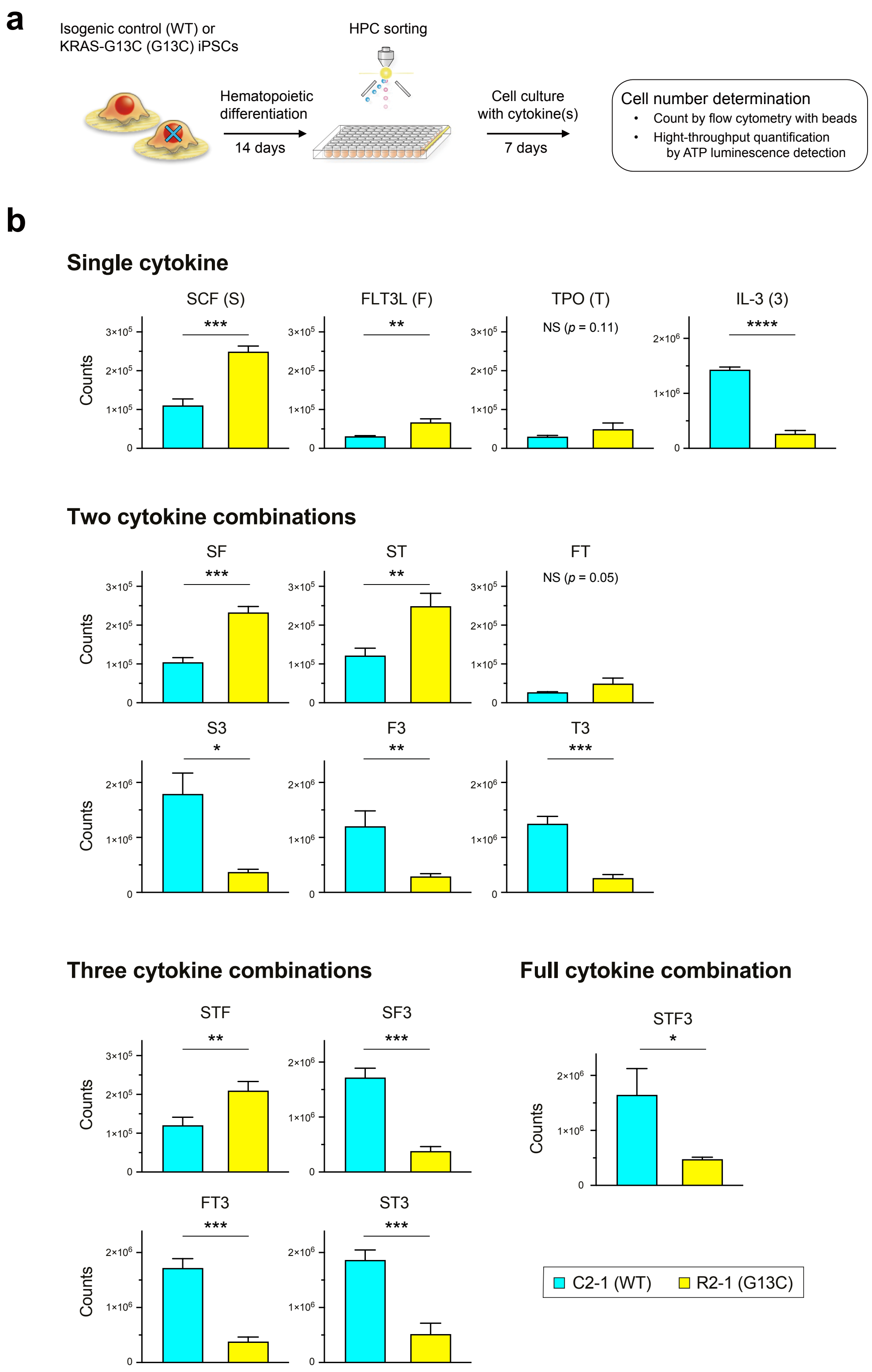

Fig. S1. Comprehensive screening of cytokine combinations to elucidate the effect of *KRAS* (G13C) mutation on iPSC-HPCs

(a) A pair of HPC samples obtained from an isogenic pair of patient 2-derived iPSCs (C2-1 and R2-1) were cultured for one week using every combination of the following cytokines as stated.
SCF (S), TPO (T), FLT3-ligand (F), and IL-3 (3).

(b) Shown are the representative cell count results determined by flow cytometry analysis using the fluorescence-beads. Mean ± SD values are shown (technicial replicates: n = 3). The statistical analysis is based on an unpaired t test.

$*p < 0.05$, $**p < 0.01$, $***p < 0.001$, $****p < 0.0001$. NS, not significant.

Figure S2

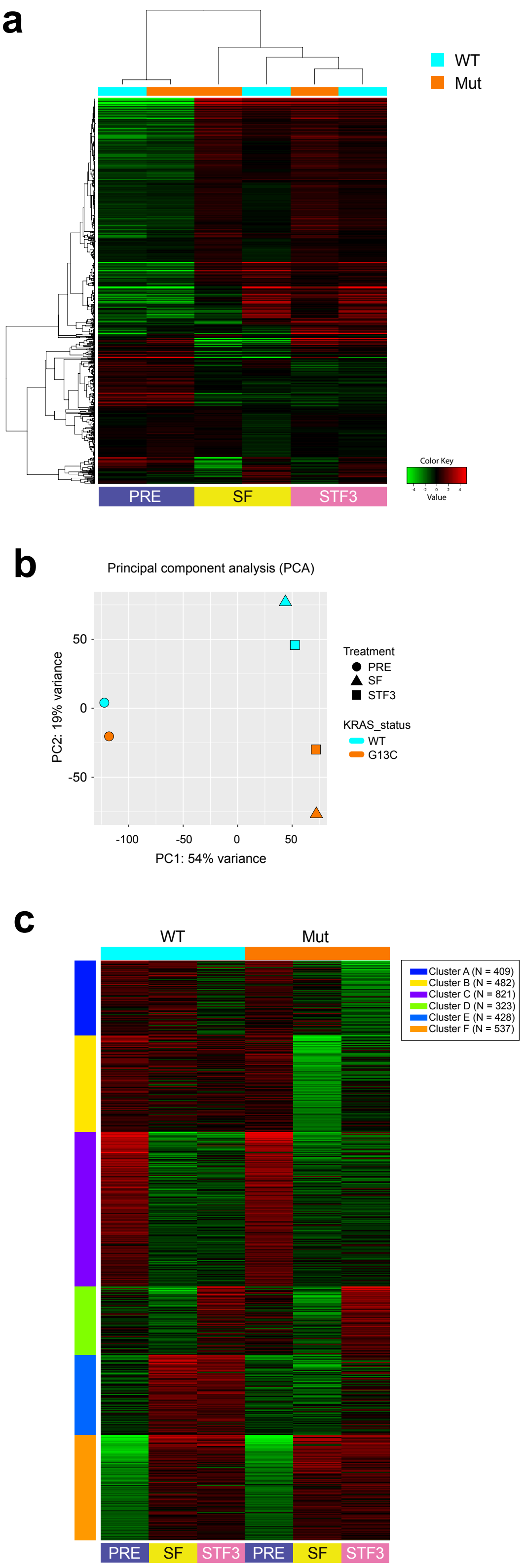

d GO Biological Process

Cluster	adj.Pval	nGenes	Pathways
A	4.5E-03	67	Nervous system development
	4.5E-03	4	Synaptic vesicle budding from presynaptic endocytic zone membrane
	9.0E-03	12	Homophilic cell adhesion via plasma membrane adhesion molecules
B	3.4E-14	119	Regulation of developmental process
	3.4E-14	135	Regulation of multicellular organismal process
	9.4E-12	55	Inflammatory response
	2.8E-11	105	Response to external stimulus
	4.1E-11	75	Cell migration
	4.1E-11	86	Positive regulation of multicellular organismal process
	5.7E-11	156	Cellular developmental process
	6.2E-11	54	Regulation of cell migration
	6.2E-11	92	Regulation of multicellular organismal development
	4.2E-10	77	Cell motility
	4.4E-10	107	Anatomical structure morphogenesis
	4.5E-10	57	Regulation of cellular component movement
	5.4E-10	52	Regulation of response to external stimulus
C	3.2E-18	97	Circulatory system development
	4.1E-16	71	Blood vessel development
	4.1E-16	73	Vasculature development
	1.0E-15	90	Tube development
	5.3E-15	78	Tube morphogenesis
	2.5E-12	59	Blood vessel morphogenesis
	3.0E-12	87	Anatomical structure formation involved in morphogenesis
	8.5E-12	55	Heart development
	1.3E-11	156	Anatomical structure morphogenesis
	1.5E-11	103	Cell adhesion
	1.5E-11	172	Cell surface receptor signaling pathway
	2.8E-11	178	Regulation of multicellular organismal process
	1.4E-10	210	Regulation of biological quality
D	1.1E-09	63	Defense response
	7.6E-09	85	Immune system process
	7.6E-09	69	Immune response
	7.6E-09	48	Leukocyte activation
	2.1E-08	37	Cytokine-mediated signaling pathway
	3.2E-08	50	Cell activation
	3.7E-08	19	Leukocyte chemotaxis
	4.1E-08	26	Leukocyte migration
	4.1E-08	9	Eosinophil migration
	7.4E-08	32	Myeloid leukocyte activation
	8.5E-08	44	Response to cytokine
	9.1E-08	42	Cellular response to cytokine stimulus
	1.2E-07	34	Inflammatory response
	2.0E-07	50	Secretion by cell
	2.3E-07	27	Granulocyte activation
E	9.4E-16	43	Extracellular structure organization
	4.6E-14	105	Response to external stimulus
	6.5E-14	53	Cell activation involved in immune response
	6.5E-14	81	Secretion by cell
	1.7E-13	52	Leukocyte activation involved in immune response
	2.4E-13	76	Cell activation
	2.6E-13	151	Cellular developmental process
	3.3E-13	45	Myeloid cell activation involved in immune response
	3.3E-13	83	Secretion
	3.7E-13	111	Regulation of localization
	6.0E-13	145	Cell differentiation
	7.6E-13	123	Immune system process
	2.7E-12	48	Myeloid leukocyte activation
	2.8E-12	68	Leukocyte activation
	5.0E-12	98	Immune response
F	1.3E-10	83	Cell activation
	3.3E-10	52	Myeloid leukocyte activation
	8.9E-10	74	Leukocyte activation
	1.1E-09	134	Regulation of multicellular organismal process
	1.3E-09	55	Regulated exocytosis
	1.9E-09	59	Exocytosis
	1.9E-08	80	Secretion by cell
	2.4E-08	42	Leukocyte degranulation
	2.4E-08	84	Secretion
	3.3E-08	42	Myeloid cell activation involved in immune response
	3.3E-08	104	Immune response
	4.1E-08	42	Myeloid leukocyte mediated immunity
	4.2E-08	72	Lipid metabolic process
	4.7E-08	48	Leukocyte activation involved in immune response

e Hallmark. MSigDB

Cluster	adj.Pval	nGenes	Pathways
A	4.6E-08	18	HALLMARK INTERFERON GAMMA RESPONSE
	2.2E-04	9	HALLMARK INTERFERON ALPHA RESPONSE
	9.4E-03	4	HALLMARK NOTCH SIGNALING
	9.4E-03	10	HALLMARK KRAS SIGNALING DN
B	5.4E-08	20	HALLMARK INFLAMMATORY RESPONSE
	9.2E-07	18	HALLMARK KRAS SIGNALING UP
	7.8E-06	11	HALLMARK IL6 JAK STAT3 SIGNALING
	5.5E-05	15	HALLMARK TNFA SIGNALING VIA NFKB
	2.1E-04	14	HALLMARK ALLOGRAFT REJECTION
	6.6E-04	13	HALLMARK IL2 STAT5 SIGNALING
	1.8E-03	12	HALLMARK HYPOXIA
	1.8E-03	12	HALLMARK INTERFERON GAMMA RESPONSE
	1.8E-03	12	HALLMARK COMPLEMENT
	1.9E-03	6	HALLMARK TGF BETA SIGNALING
	5.0E-03	11	HALLMARK MYOGENESIS
C	6.4E-06	22	HALLMARK MYOGENESIS
	4.1E-05	20	HALLMARK HEME METABOLISM
	1.3E-04	19	HALLMARK EPITHELIAL MESENCHYMAL TRANSITION
	9.2E-04	17	HALLMARK ESTROGEN RESPONSE EARLY
	9.2E-04	14	HALLMARK UV RESPONSE DN
	2.3E-03	16	HALLMARK TNFA SIGNALING VIA NFKB
	6.0E-03	15	HALLMARK HYPOXIA
D	8.6E-05	12	HALLMARK INFLAMMATORY RESPONSE
	4.4E-04	7	HALLMARK IL6 JAK STAT3 SIGNALING
	4.4E-04	10	HALLMARK INTERFERON GAMMA RESPONSE
	4.4E-04	10	HALLMARK IL2 STAT5 SIGNALING
	4.4E-04	10	HALLMARK ALLOGRAFT REJECTION
	4.4E-04	10	HALLMARK KRAS SIGNALING UP
	1.8E-03	9	HALLMARK EPITHELIAL MESENCHYMAL TRANSITION
	3.2E-03	7	HALLMARK COAGULATION
	5.7E-03	8	HALLMARK COMPLEMENT
E	2.5E-11	23	HALLMARK EPITHELIAL MESENCHYMAL TRANSITION
	6.5E-10	21	HALLMARK COMPLEMENT
	3.4E-09	20	HALLMARK MYOGENESIS
	1.4E-07	15	HALLMARK COAGULATION
	2.6E-06	16	HALLMARK TNFA SIGNALING VIA NFKB
	1.2E-05	15	HALLMARK KRAS SIGNALING UP
	5.1E-05	14	HALLMARK HYPOXIA
	1.9E-04	13	HALLMARK ADIPOGENESIS
	2.1E-03	11	HALLMARK ESTROGEN RESPONSE EARLY
	2.1E-03	11	HALLMARK ESTROGEN RESPONSE LATE
	2.1E-03	11	HALLMARK INFLAMMATORY RESPONSE
	2.1E-03	11	HALLMARK IL2 STAT5 SIGNALING
	4.4E-03	6	HALLMARK CHOLESTEROL HOMEOSTASIS
F	9.9E-11	16	HALLMARK CHOLESTEROL HOMEOSTASIS
	2.2E-07	20	HALLMARK IL2 STAT5 SIGNALING
	8.5E-07	19	HALLMARK P53 PATHWAY
	1.4E-05	17	HALLMARK TNFA SIGNALING VIA NFKB
	1.6E-05	15	HALLMARK APOPTOSIS
	4.5E-05	16	HALLMARK KRAS SIGNALING UP
	1.6E-04	15	HALLMARK ESTROGEN RESPONSE LATE
	4.0E-04	14	HALLMARK MTORC1 SIGNALING
	4.0E-04	14	HALLMARK EPITHELIAL MESENCHYMAL TRANSITION
	4.0E-04	14	HALLMARK INFLAMMATORY RESPONSE
	4.0E-04	14	HALLMARK XENOBIOTIC METABOLISM
	5.2E-04	12	HALLMARK FATTY ACID METABOLISM
	8.6E-04	9	HALLMARK ANDROGEN RESPONSE
	3.3E-03	12	HALLMARK ESTROGEN RESPONSE EARLY
	3.3E-03	12	HALLMARK MYOGENESIS

Fig. S2. Characterization of altered transcriptome profiles brought by a single *KRAS* (G13C) mutation in iPSC-derived HPCs Patient 1 samples.

Six samples from patient 1 were subjected to the iDEP program-based analysis.

(a) A heatmap demonstration with hierarchal clustering with the top 1,000 most variable genes. The remarkable similarity between genotypes (WT vs. Mut) is obvious for pre-expansion HPCs (PRE). Substantial changes upon stimulation in profiles are evident, with their difference between genotypes becoming greater.

(b) Principal component analysis (PCA) depicting a close relationship of PRE samples and distinct characteristics that become evident for control (WT) and mutant (G13C) samples after expansion cultures.

(c-e) A heatmap generated by K-means clustering with the use of 3,000 most variable genes (c). Enriched pathways were identified with reference to the pathway databases for the gene sets classified in each cluster by the functions provided with the iDEP. Shown are the result tables analyzed with the GO Biological Process (d) and the Hallmark. MSigDB pathway database (e). Note that pathways related to myeloid cell biology and immune/inflammatory responses are found enriched. The enrichment in pathways related to the KRAS signaling is also noted.of hematopoietic progenitor cells differentiated from RALD patient-derived iPSCs.

Figure S3

a

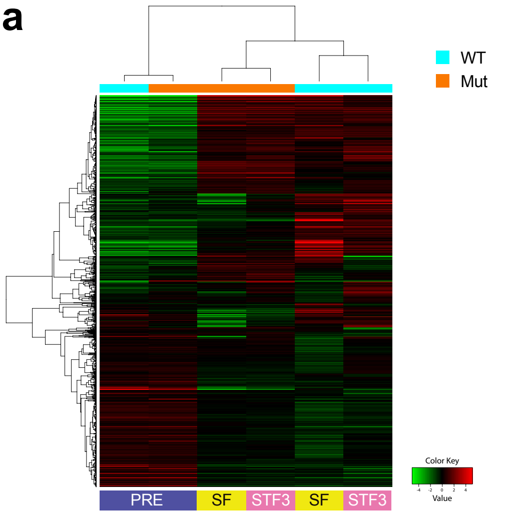

b

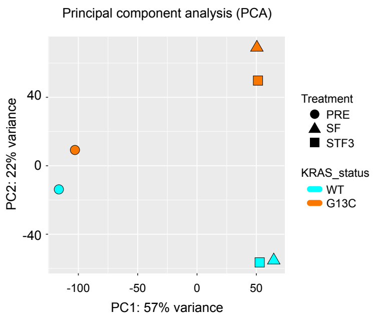

c

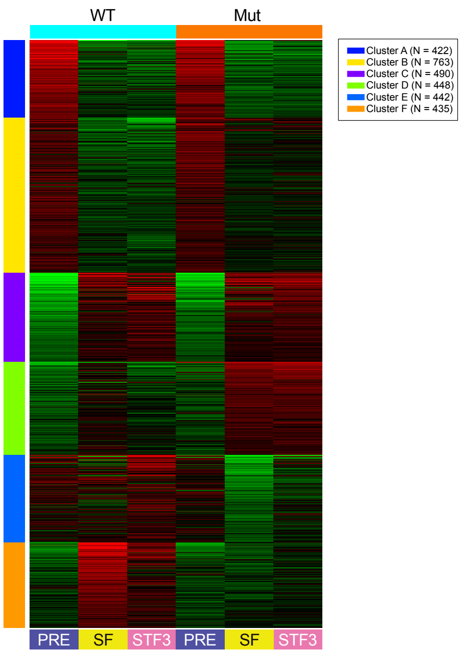

d GO Biological Process

Cluster	adj.Pval	nGenes	Pathways
A	1.8E-18	120	Anatomical structure morphogenesis
	8.9E-18	68	Tube development
	1.4E-17	68	Circulatory system development
	1.7E-17	60	Tube morphogenesis
	1.7E-17	70	Anatomical structure formation involved in morphogenesis
	1.9E-17	55	Cardiovascular system development
	3.6E-17	53	Blood vessel development
	4.4E-17	54	Vasculature development
	1.4E-14	46	Blood vessel morphogenesis
	1.5E-13	41	Angiogenesis
	8.7E-13	90	Movement of cell or subcellular component
	8.7E-13	120	Regulation of multicellular organismal process
	2.5E-12	104	Regulation of developmental process
	5.0E-12	71	Cell migration
	5.8E-12	71	Cell adhesion
B	8.1E-19	176	Anatomical structure morphogenesis
	8.1E-14	87	Circulatory system development
	1.9E-13	198	Animal organ development
	3.6E-13	74	Tube morphogenesis
	3.6E-13	84	Tube development
	1.2E-12	85	Animal organ morphogenesis
	7.5E-12	103	Cell adhesion
	6.8E-11	127	Movement of cell or subcellular component
	8.4E-11	128	Cell development
	1.4E-10	82	Anatomical structure formation involved in morphogenesis
	1.6E-10	52	Heart development
	1.7E-10	219	Cellular developmental process
	1.8E-10	59	Blood vessel development
	2.1E-10	211	Cell differentiation
C	2.3E-20	65	Myeloid leukocyte activation
	2.3E-20	90	Leukocyte activation
	2.5E-20	96	Cell activation
	4.3E-20	66	Leukocyte activation involved in immune response
	3.3E-19	57	Myeloid cell activation involved in immune response
	4.0E-18	55	Leukocyte degranulation
	7.7E-18	68	Leukocyte mediated immunity
	1.1E-17	55	Myeloid leukocyte mediated immunity
	1.9E-16	68	Exocytosis
	2.7E-16	63	Regulated exocytosis
	3.3E-15	115	Immune response
	5.0E-15	49	Granulocyte activation
	1.4E-14	48	Neutrophil activation
	1.9E-14	77	Immune effector process
D	8.9E-10	69	Cell adhesion
	8.9E-10	101	Anatomical structure morphogenesis
	1.7E-09	142	Cellular developmental process
	3.5E-09	113	Regulation of multicellular organismal process
	2.7E-08	81	Movement of cell or subcellular component
	2.7E-08	55	Cellular component morphogenesis
	2.7E-08	133	Cell differentiation
	2.8E-08	75	Locomotion
	5.0E-08	51	Cell morphogenesis
	5.1E-08	57	Negative regulation of multicellular organismal process
	5.5E-08	89	Response to external stimulus
	5.5E-08	98	Regulation of localization
	6.2E-08	104	Cell surface receptor signaling pathway
	8.3E-08	45	Regulation of cell migration
E	6.1E-12	121	Immune system process
	5.1E-10	69	Cell activation
	1.1E-09	63	Leukocyte activation
	1.6E-09	92	Response to external stimulus
	1.9E-09	74	Secretion
	1.0E-08	64	Cell adhesion
	1.0E-08	110	Cellular response to chemical stimulus
	1.0E-08	56	Cellular response to cytokine stimulus
	1.3E-08	58	Response to cytokine
	3.0E-08	71	Regulation of immune system process
	3.0E-08	89	Positive regulation of response to stimulus
	3.7E-08	131	Regulation of response to stimulus
	5.1E-08	66	Secretion by cell
	5.7E-08	125	Regulation of biological quality
F	8.0E-43	76	Granulocyte activation
	1.7E-42	75	Neutrophil activation
	2.6E-41	82	Myeloid leukocyte activation
	2.6E-41	76	Myeloid cell activation involved in immune response
	2.6E-41	73	Neutrophil activation involved in immune response
	5.0E-41	75	Leukocyte degranulation
	8.0E-41	72	Neutrophil degranulation
	1.3E-40	83	Leukocyte activation involved in immune response
	2.6E-40	72	Neutrophil mediated immunity
	1.8E-39	74	Myeloid leukocyte mediated immunity
	2.3E-35	83	Leukocyte mediated immunity
	4.9E-35	134	Immune response
	6.0E-35	80	Regulated exocytosis
	1.5E-32	82	Exocytosis

e Hallmark. MSigDB

Cluster	adj.Pval	nGenes	Pathways
A	2.1E-11	23	HALLMARK EPITHELIAL MESENCHYMAL TRANSITION
	1.9E-07	18	HALLMARK ALLOGRAFT REJECTION
	7.6E-07	17	HALLMARK MYOGENESIS
	8.2E-05	14	HALLMARK HYPOXIA
	3.0E-04	13	HALLMARK KRAS SIGNALING UP
	9.9E-04	12	HALLMARK IL2 STAT5 SIGNALING
	3.0E-03	11	HALLMARK APICAL JUNCTION
	3.0E-03	11	HALLMARK GLYCOLYSIS
	7.7E-03	10	HALLMARK INTERFERON GAMMA RESPONSE
	7.7E-03	10	HALLMARK COMPLEMENT
	7.7E-03	10	HALLMARK INFLAMMATORY RESPONSE
	8.9E-03	8	HALLMARK UV RESPONSE DN
	8.9E-03	4	HALLMARK ANGIOGENESIS
B	6.0E-11	29	HALLMARK EPITHELIAL MESENCHYMAL TRANSITION
	1.7E-04	8	HALLMARK ANGIOGENESIS
	3.1E-04	18	HALLMARK INFLAMMATORY RESPONSE
	3.1E-04	18	HALLMARK HEME METABOLISM
	5.9E-04	17	HALLMARK HYPOXIA
	5.9E-04	17	HALLMARK ESTROGEN RESPONSE LATE
	5.9E-04	17	HALLMARK KRAS SIGNALING UP
	1.1E-03	16	HALLMARK TNFA SIGNALING VIA NFKB
	1.1E-03	10	HALLMARK IL6 JAK STAT3 SIGNALING
	1.1E-03	16	HALLMARK ESTROGEN RESPONSE EARLY
	1.1E-03	16	HALLMARK MYOGENESIS
	1.1E-03	13	HALLMARK UV RESPONSE DN
	1.1E-03	13	HALLMARK COAGULATION
	1.8E-03	6	HALLMARK HEDGEHOG SIGNALING
	6.7E-03	14	HALLMARK GLYCOLYSIS
C	5.8E-07	19	HALLMARK IL2 STAT5 SIGNALING
	1.3E-05	15	HALLMARK APOPTOSIS
	2.9E-05	16	HALLMARK INFLAMMATORY RESPONSE
	9.2E-05	15	HALLMARK TNFA SIGNALING VIA NFKB
	2.8E-04	14	HALLMARK COMPLEMENT
	2.8E-04	14	HALLMARK EPITHELIAL MESENCHYMAL TRANSITION
	8.2E-04	13	HALLMARK ESTROGEN RESPONSE LATE
	8.2E-04	13	HALLMARK P53 PATHWAY
	2.5E-03	7	HALLMARK CHOLESTEROL HOMEOSTASIS
	2.9E-03	8	HALLMARK ANDROGEN RESPONSE
	6.7E-03	9	HALLMARK UV RESPONSE DN
	6.7E-03	11	HALLMARK KRAS SIGNALING UP
D	4.1E-08	14	HALLMARK INTERFERON ALPHA RESPONSE
	2.3E-07	18	HALLMARK TNFA SIGNALING VIA NFKB
	9.0E-07	17	HALLMARK INTERFERON GAMMA RESPONSE
	2.1E-05	15	HALLMARK KRAS SIGNALING UP
	6.7E-05	14	HALLMARK EPITHELIAL MESENCHYMAL TRANSITION
	6.7E-05	14	HALLMARK INFLAMMATORY RESPONSE
	1.5E-04	11	HALLMARK COAGULATION
	2.2E-04	13	HALLMARK P53 PATHWAY
	8.2E-04	10	HALLMARK UV RESPONSE DN
	2.8E-03	11	HALLMARK HYPOXIA
	5.6E-03	6	HALLMARK CHOLESTEROL HOMEOSTASIS
	7.0E-03	10	HALLMARK ESTROGEN RESPONSE EARLY
	7.0E-03	10	HALLMARK IL2 STAT5 SIGNALING
	7.0E-03	10	HALLMARK KRAS SIGNALING DN
E	4.9E-06	11	HALLMARK IL6 JAK STAT3 SIGNALING
	4.9E-06	16	HALLMARK KRAS SIGNALING UP
	1.3E-05	15	HALLMARK IL2 STAT5 SIGNALING
	1.3E-05	15	HALLMARK ALLOGRAFT REJECTION
	2.0E-04	13	HALLMARK INTERFERON GAMMA RESPONSE
	2.0E-04	13	HALLMARK INFLAMMATORY RESPONSE
	7.0E-04	12	HALLMARK COMPLEMENT
	2.2E-03	11	HALLMARK TNFA SIGNALING VIA NFKB
	2.2E-03	11	HALLMARK ESTROGEN RESPONSE LATE
	6.0E-03	10	HALLMARK HYPOXIA
	6.0E-03	10	HALLMARK ESTROGEN RESPONSE EARLY
	6.0E-03	10	HALLMARK EPITHELIAL MESENCHYMAL TRANSITION
F	5.0E-12	23	HALLMARK INFLAMMATORY RESPONSE
	3.8E-07	17	HALLMARK COMPLEMENT
	1.5E-06	16	HALLMARK TNFA SIGNALING VIA NFKB
	3.6E-05	14	HALLMARK KRAS SIGNALING UP
	6.0E-04	12	HALLMARK IL2 STAT5 SIGNALING

Fig. S3. Characterization of altered transcriptome profiles brought by a single *KRAS* (G13C) mutation in iPSC-derived HPCs (patient 2 samples), related to Figures 2 and 3.
Six samples from patient 2 were subjected to the iDEP program-based analysis.
(a) A heatmap demonstration with hierarchal clustering with the top 1,000 most variable genes. The remarkable similarity between genotypes (WT vs. Mut) is obvious for pre-expansion HPCs (PRE). Substantial changes upon stimulation in profiles are evident, with their difference between genotypes becoming greater.
(b) Principal component analysis (PCA) depicting a close relationship of PRE samples and distinct characteristics that become evident for control (WT) and mutant (G13C) samples after expansion cultures.
(c-e) A heatmap generated by K-means clustering with the use of 3,000 most variable genes (c). Enriched pathways were identified with reference to the pathway databases for the gene sets classified in each cluster by the functions provided with the iDEP. Shown are the result tables analyzed with the GO Biological Process (d) and the Hallmark. MSigDB pathway database (e). Note that pathways related to myeloid cell biology and immune/inflammatory responses are found enriched. The enrichment in pathways related to the KRAS signaling is also noted.of hematopoietic progenitor cells differentiated from RALD patient-derived iPSCs.

Figure S4

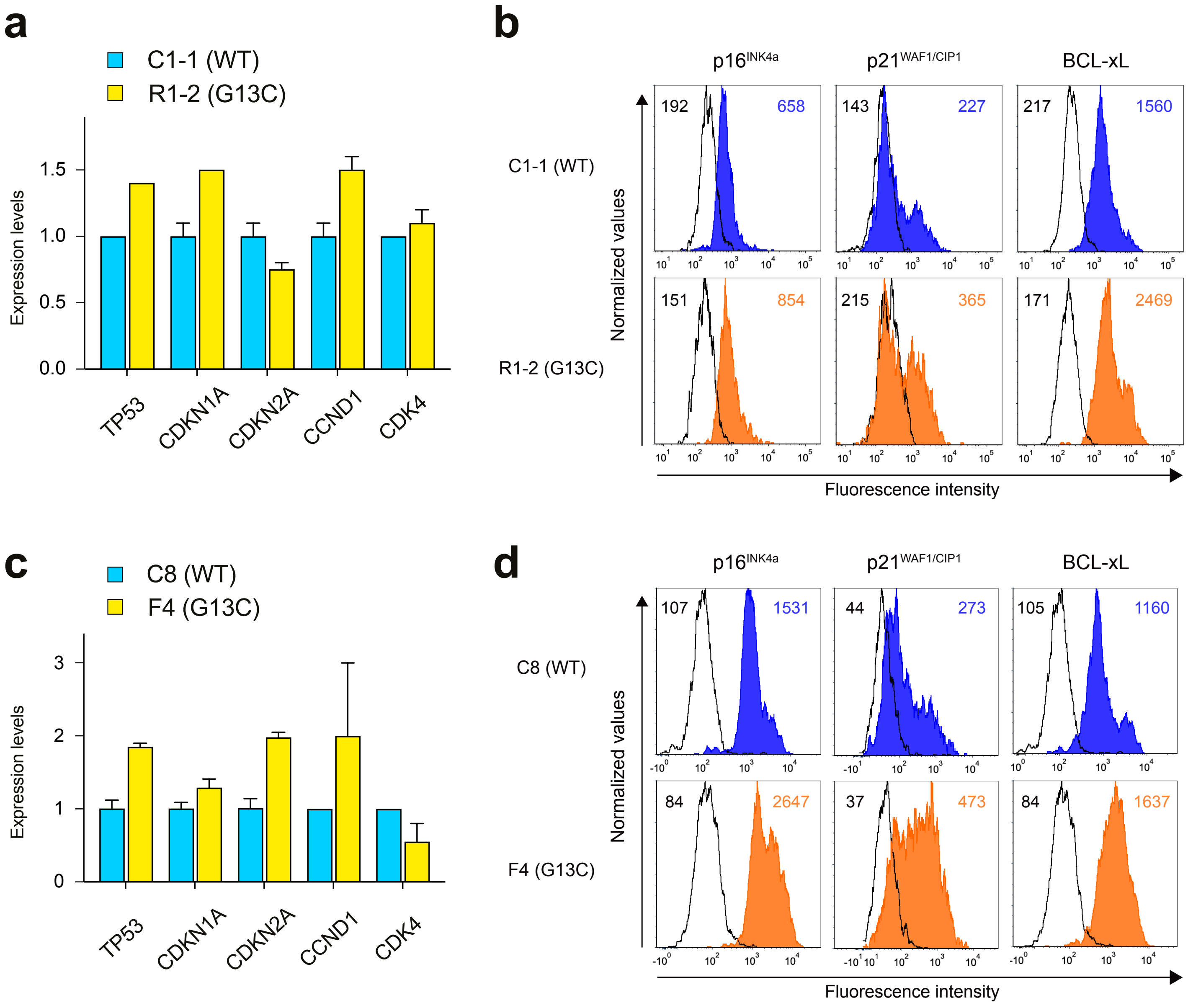

Fig. S4. Altered gene and protein expression of cell-cycle and apoptosis-related molecules in *KRAS*-mutant HPCs, related to Figure 4.

The analysis similar to that shown in Figure 4 was conducted with another isogenic pair of control (WT) and mutant (G13C) iPSCs, both derived from patient 1: C1-1 vs. R1-2 (primarily established clones) and C8 vs. F4 (gene-corrected vs. pre-correction mutant clones, originated from R1-2).

(a and c) Quantitative RT-PCR analysis of selected genes related to the regulation of cell cycle and apoptosis in expanded HPCs under standard conditions. The representative results obtained with each iogenic pair in two independent experiments are presented as mean ± SEM from biological duplicate samples.

(b and d) Intracellular flow cytometry analysis for the expression levels of indicated molecules in cultured HPCs. Shown are the representative overlay plots. Mean fluorescent values are shown for

Figure S5

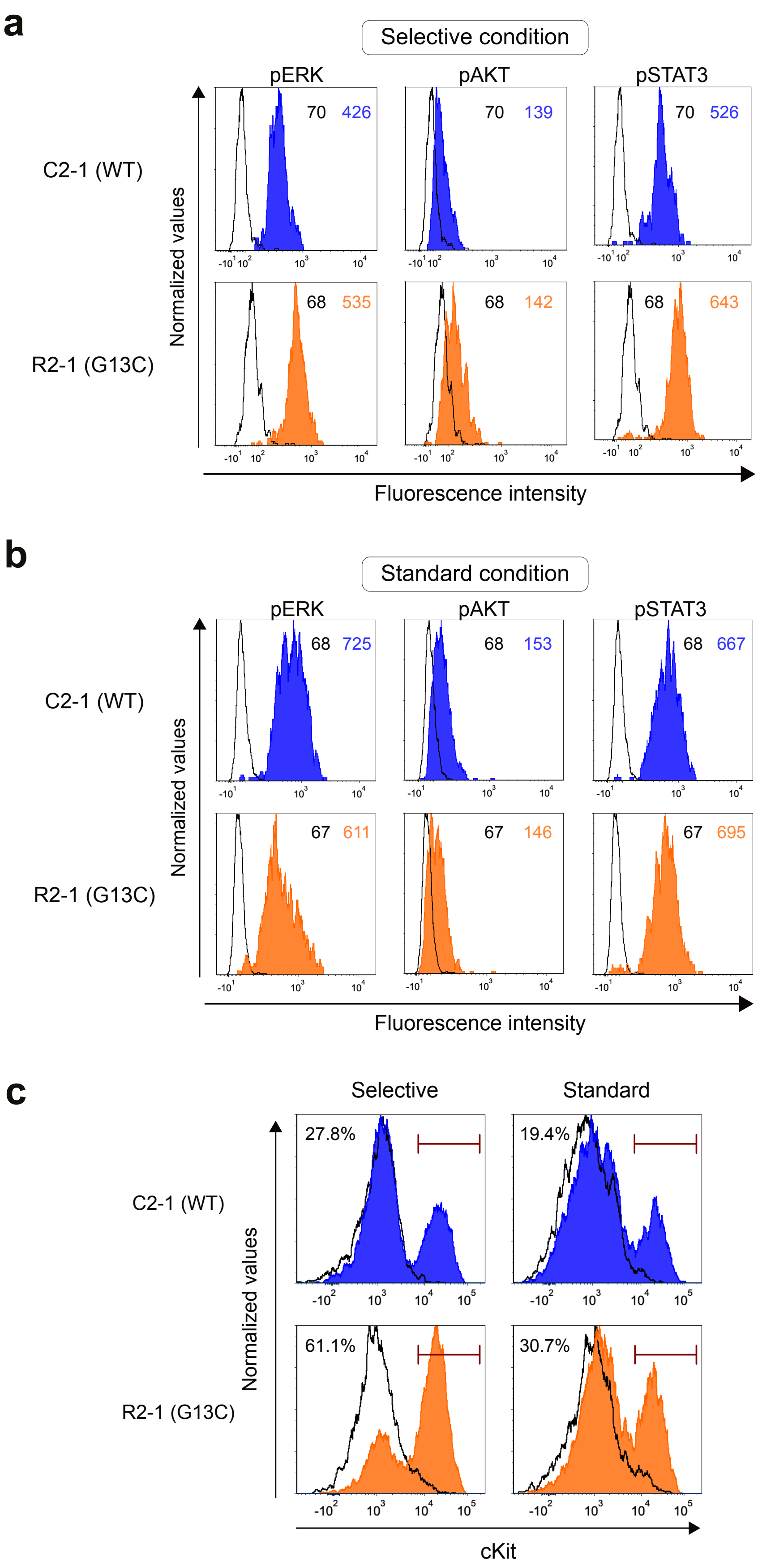

Fig. S5. Aberrancies in cell signaling pathways and cKit expression conferred on cultured iPSC-derived HPCs carrying the *KRAS* (G13C) mutation, related to Figure 5.

(a and b) Signaling events in expanded HPCs were determined by intracellular expression of pERK, pAKT, and pSTAT3, three well-known pathway molecules acting downstream of RAS, by flow cytometry analysis. Used are the isogenic pair derived from patient 2, C2-1 (WT) and R2-1 (G13C). HPCs were cultured before analysis either with Selective- (a) or Standard- (b) condition. Open histograms represent isotype antibody-control, while closed histograms show the specific staining for indicated molecules. Numbers indicate the mean fluorescence intensity.

(c) Cell surface expression levels of cKit determined by flow cytometry analysis. The isogenic pair of C2-1 and R2-1 was tested after expansion cultures. Open histograms, isotype-control; closed histograms, cKit-staining. The percent positive values are shown.

Figure S6

a

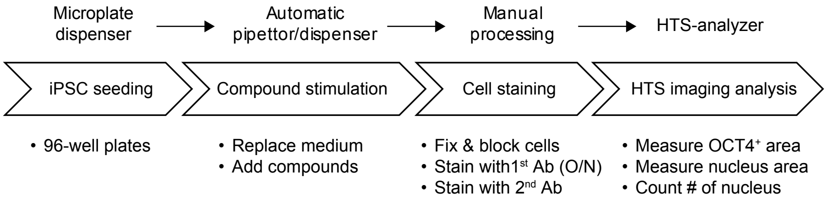

b

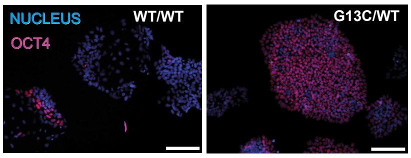

c

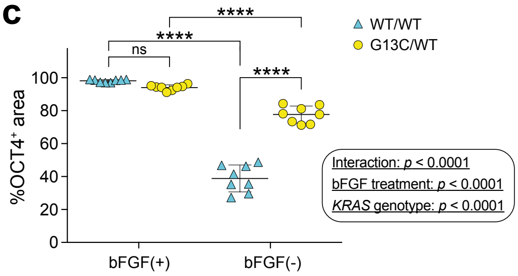

d

Mechanism	No of hits
MEK (MAPKK)	7
FGF receptor	6
Bromodomain (BRD/CBP/p300)	6
TGF-β receptor	5
GSK3-β	5
Notch/γ-secretase	3
ERK (MAPK)	2
RAF (MAPKKK)	2
p90RSK	1
GABAa receptor agonist	1
mGluR2 PAM	1
Histone methyltransferase (HMT)	1
Total	40

e

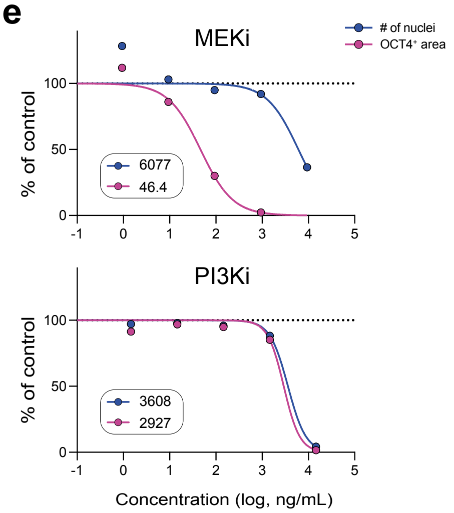

f

GPCRs	Ion channels	Transporters	Nuclear receptors	Kinases	Proteases	Other enzymes	Others	Total
174	109	38	18	231	12	147	177	906

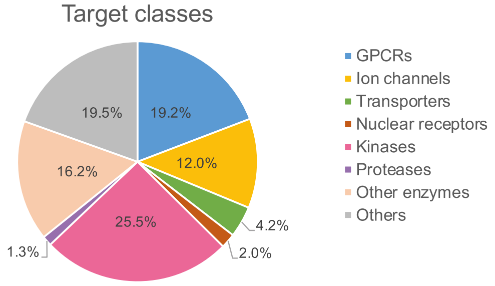

Fig. S6. Inhibitor library screening to identify effective modulators of aberrant KRAS signaling in iPSCs
(a) Schematic representation of an iPSC-based screening flow. iPSCs carrying the *KRAS*(G13C) mutation (R1-2) were seeded and maintained in 96-well plates using semi-automated processes. Test compounds were added on day 1 using an automatic pipettor/dispenser. Cells were cultured in the absence of bFGF, fixed and permeabilized on day 5, followed by overnight staining with the anti-OCT4 antibody. High-throughput screening (HTS) imaging analysis was performed on day 6 after being stained with the fluorescence-conjugated secondary antibody and Hoechst 33342.
(b) Immunohistochemistry of iPSCs cultured for 5 days in the absence of bFGF. The images show that while most control cells (C1-1, WT/WT) lose OCT4 expression (left), KRAS-mutant iPSCs (R1-2, G13C/WT) are still expressing the pluripotent marker (right). Scale bars: 100 μm.
(c) Quantitative characterization for the ability specific to iPSCs carrying the *KRAS(G13C)* mutation, defined as “enforced retention of self-renewal.” The percentages of nucleated cells that expressed OCT4 were quantified using the isogenic pair of iPSCs (C1-1 and R1-2). Mean ± SD values are shown (experimental replicates: n = 8). The *P* values are shown based on the statistical analysis carried out with 2-way ANOVA, followed by Tukey’s multiple comparisons test. ****p < 0.0001; ns, not significant. Note that a majority of mutant iPSCs (77.7%) remain viable while expressing OCT4 in the absence of bFGF, reflecting the *KRAS (G13C)*-mediated abnormality that potentially serves as “druggable target.”
(d and e) A summary of screening results testing a 906 bioactive compound library. We sought to look for the ability to reverse the abnormality mentioned above (i.e., the effect to induce loss of OCT4 expression) without exhibiting significant cell toxicity.
(d) Results shown in a table as 40 “hits” with their mode of action (MOA, Mechanism) and the number of compounds contained in each category. (e) Shown are representative examples of a “hit” (top, MEK inhibition) and a “non-hit” (bottom, PI3K inhibition). The IC_{50} values calculated from plotted reverse sigmoid curves are shown.
(f) MoA target classification of the compound library used in the screening experiments.

Figure S7

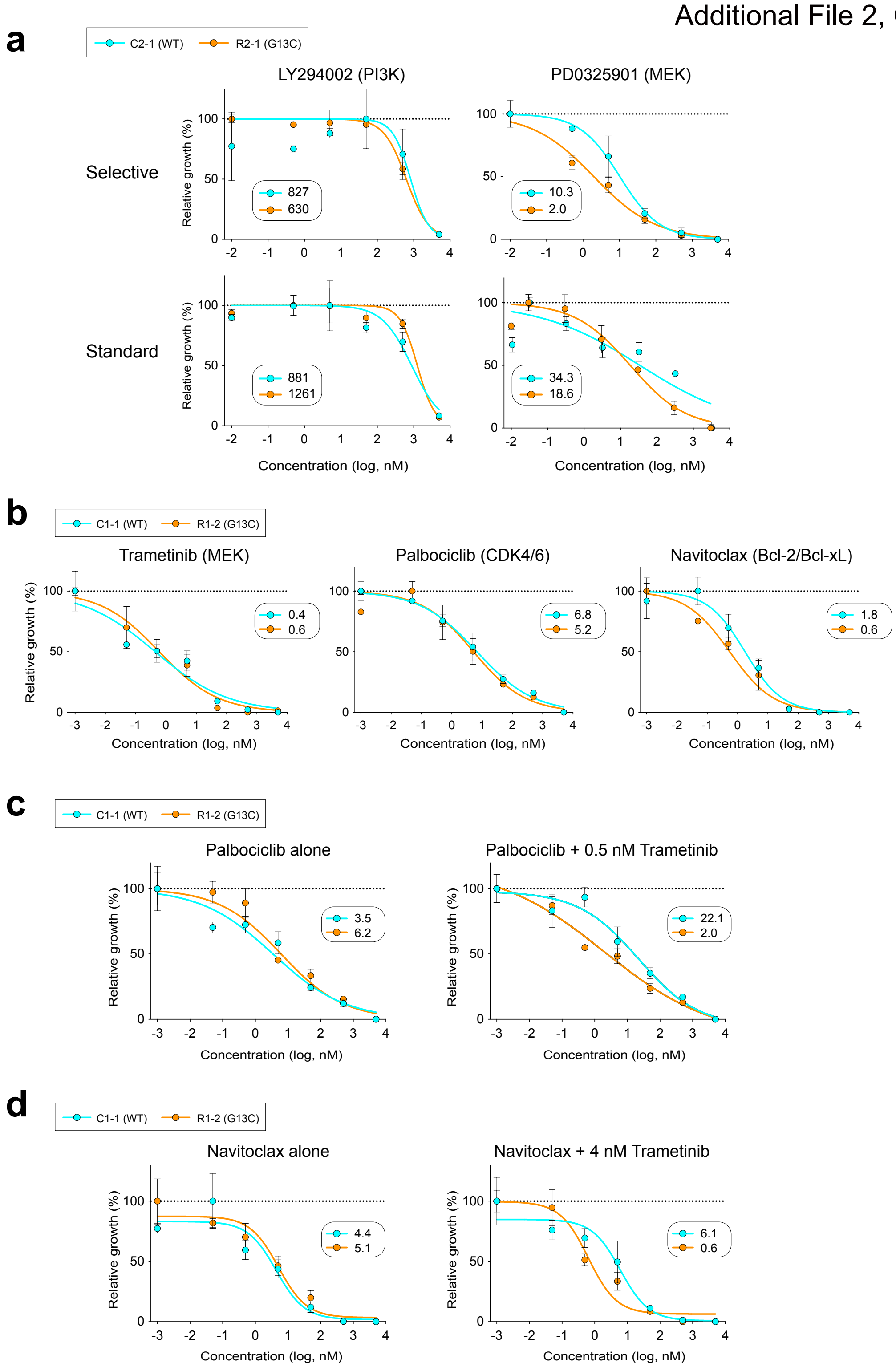

Fig. S7. Utilization of the established drug screening platform to explore inhibitory effects significantly selective on *KRAS*-mutant HPCs, related to Figures 6 and 7.

(a) Validation of the screening system with the well-characterized modulators of signaling pathways downstream of KRAS. Dose-response plots assessing growth inhibitory effects of a single reagent on either control (C2-1, blue) or *KRAS*-mutant (R2-1, orange) HPCs cultured in 2 distinct cytokine conditions (Selective or Standard). The percent growth was estimated relative to the control values (DMSO) with the ATP-measurement-based viability assay (technical replicates: n = 3). Calculated IC_{50} values are indicated. Note the absence of selective inhibitory effects for the PI3K inhibitor LY294002 in both conditions. In contrast, the MEK inhibitor PD0325901 exhibits modest selectivity towards the mutant cells. Because the Selective condition (SCF and FLT3L only) often led to highly variable, non-informative outcomes due to overall poor cell viability, we decided to use only Standard culture for the subsequent experiments to ensure reliable consequences.

(b) Dose-response curves obtained with a patient 1-derived isogenic pair of iPSC-derived HPCs (C1-1 and R1-2) treated by a single reagent (technical replicates: n = 3). IC_{50} values are shown. Note that the modest selective activity is observed for Navitoclax, whereas no evident selectivity is seen for Trametinib and Palbociclib.

(c and d) Potential enhancement of selective inhibitory effects on *KRAS*-mutant HPCs by combination treatment. Growth inhibitory effects were compared between single- and combined-treatments for the pair of C1-1 and R1-2 (technical replicates: n = 3).

(c) Palbociclib dose-responses either alone (left) or in the presence of a low dose Trametinib (right). The IC_{50} value for R1-2 samples gets lower (2.0 nM) in the presence of 0.5 nM Trametinib compared with that obtained with Palbociclib alone (6.2 nM), whereas it is not the case for C1-1 samples (3.5 nM with Palbociclib alone and 22.1 nM in combination).

(d) Navitoclax dose-responses either alone (left) or in the presence of a low dose Trametinib (right). The IC_{50} value for R1-2 samples becomes lower (0.6 nM) in the presence of 4 nM Trametinib compared with that obtained with Navitoclax alone (5.1 nM), whereas the values remain unaltered for C1-1 samples (4.4 nM with Navitoclax alone and 6.1 nM in combination).
